# Postcardiotomy extracorporeal membrane oxygenation in patients older than 70 years: Characteristics, outcomes, and variables associated with mortality

**DOI:** 10.1016/j.xjon.2025.04.004

**Published:** 2025-05-03

**Authors:** Maged Makhoul, Silvia Mariani, Bas C.T. van Bussel, Dominik Wiedemann, Diyar Saeed, Michele Di Mauro, Matteo Pozzi, Luca Botta, Udo Boeken, Robertas Samalavicius, Karl Bounader, Xiaotong Hou, Jeroen J.H. Bunge, Hergen Buscher, Leonardo Salazar, Bart Meyns, Michael A. Mazzeffi, Marco L. Sacha Matteucci, Sandro Sponga, Graeme MacLaren, Claudio Russo, Francesco Formica, Pranya Sakiyalak, Antonio Fiore, Daniele Camboni, Giuseppe Maria Raffa, Rodrigo Diaz, I-wen Wang, Jae-Seung Jung, Jan Belohlavek, Vin Pellegrino, Giacomo Bianchi, Matteo Pettinari, Alessandro Barbone, José P. Garcia, Kiran Shekar, Glenn Whitman, Gil Bolotin, Roberto Lorusso, PELS-1 Investigators

**Affiliations:** a Cardio-Thoracic Surgery Department, and Cardiovascular Research Institute Maastricht, Maastricht, The Netherlands; b Cardiac Surgery Department, Rambam Medical Centre, Haifa, Israel; c Cardiac Surgery Unit, Cardio-Thoracic and Vascular Department, Fondazione IRCCS San Gerardo dei Tintori, Monza, Italy; d Department of Intensive Care Medicine, and Cardiovascular Research Institute Maastricht (CARIM), Maastricht, The Netherlands; e Care and Public Health Research Institute, Maastricht University, Maastricht, The Netherlands; f Department of Cardiac Surgery, Medical University of Vienna, Vienna, Austria; g Department of Cardiac Surgery, Karl Landsteiner University, University Clinic St Pölten, St Pölten, Austria; h Heart Center Niederrhein, Helios Hospital Krefeld, Krefeld, Germany; i Department of Cardiac Surgery, Louis Pradel Cardiologic Hospital, Lyon, France; j Division of Cardiac Surgery, IRCCS Azienda Ospedaliero-Universitaria di Bologna, Bologna, Italy; k Department of Cardiac Surgery, Medical Faculty, Heinrich Heine University, Duesseldorf, Germany; l II Department of Anesthesiology, Centre of Anesthesia, Intensive Care and Pain Management, Vilnius University Hospital Santariskiu Klinikos, Vilnius, Lithuania; m Division of Cardiothoracic and Vascular Surgery, Pontchaillou University Hospital, Rennes, France; n Center for Cardiac Intensive Care, Beijing Institute of Heart, Lung, and Blood Vessels Diseases, Beijing Anzhen Hospital, Capital Medical University, Beijing, China; o Department of Cardiology, Erasmus MC, Rotterdam, The Netherlands; p Department of Intensive Care Adults, Erasmus MC, Rotterdam, The Netherlands; q Department of Intensive Care Medicine, St Vincent's Hospital, Sydney, Australia; r Department of Cardiology, Fundación Cardiovascular de Colombia, Bucaramanga, Colombia; s Department of Cardiac Surgery, University Hospitals Leuven and Department of Cardiovascular Sciences, University of Leuven, Leuven, Belgium; t Departments of Medicine and Surgery, University of Maryland, Baltimore, Md; u SOD Cardiochirurgia Ospedali Riuniti 'Umberto I - Lancisi - Salesi' Università Politecnica delle Marche, Ancona, Italy; v Division of Cardiac Surgery, Cardiothoracic Department, University Hospital of Udine, Udine, Italy; w Cardiothoracic Intensive Care Unit, National University Hospital, Singapore; x Cardiac Surgery Unit, Cardiac Thoracic and Vascular Department, Niguarda Hospital, Milan, Italy; y Department of Medicine and Surgery, University of Parma, Parma, Italy; z Division of Cardiovascular and Thoracic Surgery, Department of Surgery, Faculty of Medicine Siriraj Hospital, Mahidol University, Bangkok, Thailand; aa Department of Cardio-Thoracic Surgery, University Hospital Henri-Mondor, Créteil, Paris, France; bb Department of Cardiothoracic Surgery, University Medical Center Regensburg, Regensburg, Germany; cc Cardiac Surgery Unit, Department of Precision Medicine in Medical Surgical and Critical Area (Me.Pre.C.C.), University of Palermo, Palermo, Italy; dd Department for the Treatment and Study of Cardiothoracic Diseases and Cardiothoracic Transplantation, IRCCS-ISMETT (Istituto Mediterraneo per i Trapianti e Terapie ad Alta Specializzazione), Palermo, Italy; ee ECMO Unit, Departamento de Anestesia, Clínica Las Condes, Las Condes, Santiago, Chile; ff Division of Cardiac Surgery, Memorial Healthcare System, Hollywood, Fla; gg Department of Thoracic and Cardiovascular Surgery, Korea University Anam Hospital, Seoul, South Korea; hh 2nd Department of Internal Medicine, Cardiovascular Medicine General Teaching Hospital and 1st Faculty of Medicine, Charles University in Prague, Prague, Czech Republic; ii Intensive Care Unit, The Alfred Hospital, Melbourne, Victoria, Australia; jj Ospedale del Cuore Fondazione Toscana "G. Monasterio", Massa, Italy; kk Department of Cardiovascular Surgery, Ziekenhuis Oost-Limburg, Genk, Belgium; ll Cardiac Surgery Unit, IRCCS Humanitas Research Hospital, Rozzano, Milan, Italy; mm IU Health Advanced Heart & Lung Care, Indiana University Methodist Hospital, Indianapolis, Ind; nn Adult Intensive Care Services, The Prince Charles Hospital, and UQ Northside Clinic Unite, Faculty of Medicine, The University of Queensland, Brisbane, Australia; oo Cardiac Intensive Care Unit, Johns Hopkins Hospital, Baltimore, Md

**Keywords:** mechanical circulatory support, extracorporeal membrane oxygenation, postcardiotomy cardiogenic shock, cardiac surgery, age, mortality

## Abstract

**Objectives:**

Age is the main determinant for mortality in patients requiring postcardiotomy extracorporeal membrane oxygenation (PC-ECMO), but strategies to reverse this trend are unknown. This study investigates PC-ECMO outcomes in older patients (≥70 years) compared with younger patients (<70 years).

**Methods:**

This retrospective study included patients who required PC-ECMO between 2000 and 2020. Variables independently associated with in-hospital mortality were identified using mixed Cox proportional hazards models.

**Results:**

The study included 2057 patients (mean age: 62.3 [first and third quartile: 19-94]; male patients: n = 1213 [59%]): 1376 (67%) were <70 years and 680 (33%) were ≥70 years old. Older patients had more preoperative comorbidities, whereas younger patients had lower cardiac function and more preoperative intubation and vasopressor use. In-hospital mortality was 56.3% (n = 775) and 68.8% (n = 468) in the <70 year and ≥70 year groups, respectively (*P* < .001). The 7-year postdischarge survival rate was greater for the younger patient group (*P* < .001). Variables associated with in-hospital mortality in older patients were previous stroke (hazard ratio [HR], 1.39; 95% confidence interval [CI], 1.05-1.84), preoperative right ventricular failure (HR, 1.45; 95% CI, 1-2.1), aortic surgery (HR 1.65; 95% CI, 1.2-2.2), and postoperative complications including bleeding (HR 1.24; 95% CI, 1.0-1.5), cardiac arrest (HR, 1.65; 95% CI, 1.3-2.1), and right ventricular failure (HR, 1.29; 95% CI, 1.0-1.6).

**Conclusions:**

PC-ECMO mortality is high in older patients. Preoperative factors including previous stroke and right ventricular failure and postoperative factors including bleeding, cardiac arrest, and right ventricular failure should be targeted to reduce in-hospital mortality after appropriate initial selection in older patients.

## Graphical Abstract

**Figure:**
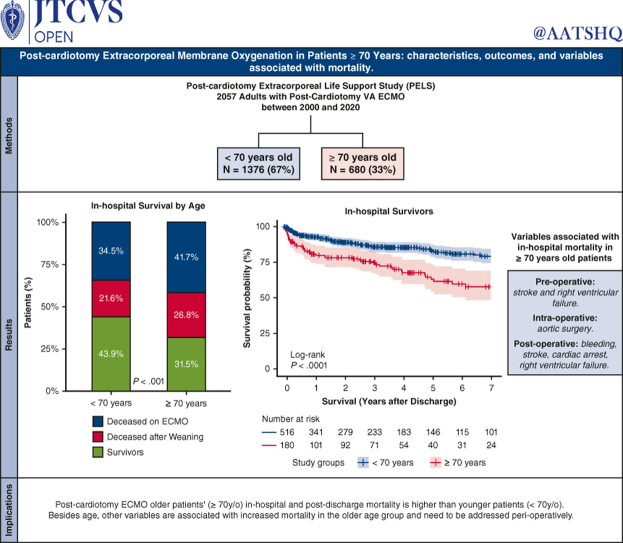

**Figure:**
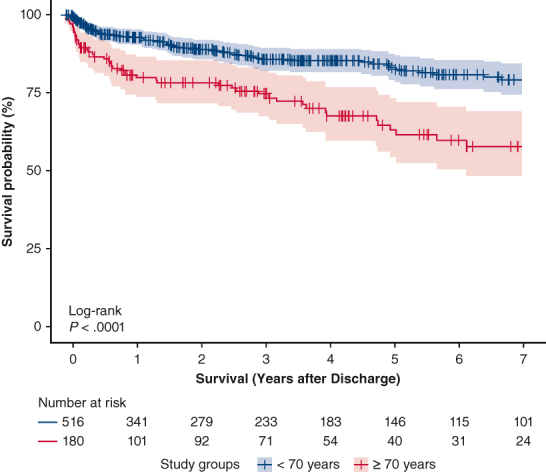
Kaplan-Meier curve illustrates the postdischarge mortality for patients who received PC-ECMO.

**Central Message**

Postcardiotomy ECMO mortality is high in elderly patients. Besides age itself, comorbidities and complications play a major role in negative outcomes.

**Perspective**

The extent of surgical treatment and ECMO support is subject to question in elderly patients because of the known high morbidity and mortality rates. Our findings showed that besides age, preoperative stroke and right ventricular failure, aortic surgery, and postoperative complications are directly associated with increased mortality in patients ≥70 years old.

Technological advancements and the development of interventional and transcatheter techniques have introduced less-invasive approaches to treat cardiac disease in patients who are older and fragile.^1^^,^^2^ Nevertheless, patients in these high-risk categories still represent a large group referred for cardiac operations.^3^ Their increased surgical and anesthesiologic complexity encompasses the need for hemodynamic support in up to 20% of patients.^4^ In some cases, inotropes and vasopressors are insufficient to support cardiac output, and mechanical circulatory support such as extracorporeal membrane oxygenation (ECMO) is required.^5^

A dilemma arises when ECMO is necessary for older patients because advanced age has been identified as one of the main determinants of mortality in almost all ECMO applications, including the postcardiotomy field, where mortality may be as high as 60%.^3^^,^^6^ The current increase in the number of patients who are old and/or fragile requiring cardiac surgery is escalating this dilemma because more and more physicians face the difficult decision of whether to support patients who are old with ECMO after surgery. This topic has been addressed only in small cohort or subgroup analyses, and more robust tools to answer this question are lacking.^7^^,^^8^ Thus, it is necessary to further investigate ECMO in-hospital and postdischarge outcomes in older patients to better understand how to maximize resources while providing optimal care to this specific population.

This study aimed to describe age-stratified characteristics, in-hospital outcomes, and postdischarge survival of patients undergoing cardiac surgery and requiring venoarterial ECMO. We hypothesized that older patients would be burdened by greater postoperative morbidity and mortality. Furthermore, we aimed to identify variables associated with mortality in patients who are older to provide tools for better patient selection, management, and outcomes.

## Methods

### Study Design

The current study is a secondary analysis of the Post-cardiotomy Extra-Corporeal Life Support (PELS) study (ClinicalTrials.gov: NCT03857217), an international, multicenter, retrospective observational study including 34 centers from 16 countries. Institutional review board approval was obtained (MUMC+, institutional review board approval number: METC-2018-0788, December 19, 2018). The need for informed consent was waived on the basis of the retrospective nature of the study, the emergency of the performed procedure, and the pseudonymization of shared data. Data were collected centrally according to data-sharing agreements between participating centers and will be shared on reasonable request to the corresponding author with the permission of all PELS-1 participating centers.

### Patient Population

Adults (≥18 years old) were included if they underwent ECMO implantation during or after a cardiac operation between January 2000 and December 2020. Exclusion criteria were ECMO after discharge or before surgery, after noncardiac operations, or not during the same cardiac surgery hospitalization. Further exclusion criteria included missing data on age or primary outcome and need for venovenous ECMO.

Patients were stratified according to their age at the moment of the first cardiac operation: patients younger than 70 years old (<70) and patients 70 years old and older (≥70), on the basis of studies showing that mortality increases significantly after the age of 70 years in patients who receive postcardiotomy extracorporeal membrane oxygenation (PC-ECMO).^9^^,^^10^

### Data Collection and Outcomes

Demographics, preoperative features, procedural characteristics, ECMO details, in-hospital morbidity and mortality, and postdischarge survival were collected in a dedicated electronic case report form (data.castoredc.com), according to the predefined protocol.^6^ Follow-up was performed through medical records review or contact with patients at the discretion of the treating center. The primary outcome of interest was all-cause, in-hospital mortality. Secondary outcomes included in-hospital complications (Online Data Supplement) and postdischarge mortality for hospital survivors.

### Statistical Analysis

Data were merged and analyzed using SPSS 26.0 (IBM Corp), and R 4.1.2 (R Foundation for Statistical Computing). The full cohort was categorized into 2 study groups (<70 years, ≥70 years) for comparison. Missing data analysis (Online Data Supplement) was conducted with the mice: Multivariate Imputation by Chained Equations R package.^11^

Descriptive statistics were conducted on available data only, and no imputations were performed for this purpose. Normality was investigated with Kolmogorov-Smirnov, Shapiro-Wilk, and inspection of histograms and Q-Q plots as appropriate. Demographic and clinical variables are expressed as numbers (valid percent on available data, excluding missing values) for categorical variables and median (first and third quartile) for continuous variables. Categorical data were compared between groups with Pearson χ^2^ or Fisher exact test. Continuous variables were analyzed using the independent-samples *t* test or Mann-Whitney *U* test, as appropriate. A Cox regression restricted cubic spline model was employed to investigate the association between age and in-hospital mortality.

Survival was investigated with the Kaplan-Meier method, and comparisons were performed with the log-rank test (survival and survminer R packages). Patients lost at follow-up were included and considered censored at the time of their last control (Online Data Supplement). Curves were truncated when the number of patients at risk from the study groups dropped to less than 10% of the initial sample.

A subgroup analysis was conducted to investigate characteristics and outcomes comparing in-hospital survivors and in-hospital nonsurvivors among patients ≥70 years. To estimate the associations between variables and in-hospital mortality in patients aged ≥70 years old, we conducted a mixed-effects Cox proportional hazards regression, using the Coxme: Mixed Effects Cox Models R package. The random effect was used to account for the dependency of observations as the result of clustering in centers and years.^6^^,^^12^ On the basis of clinical practice and literature, we first estimated a crude model, which was subsequently adjusted for sets of variables deemed potential confounders for the association with mortality. The mixed-effects Cox proportional hazards models were developed on 5 imputed datasets (mice: Multivariate Imputation by Chained Equations R package)^11^ created with “cart” method. Mixed-Cox models were run on each of these datasets, and results were pooled (junkka/ehahelper: Helper Functions for Event History Analysis R package) to obtain estimates as hazard ratios (HRs) with their 95% confidence intervals (CIs) and *P* values.

A further subgroup analysis was conducted on patients >80 years. On the basis of the possible variations in extracorporeal life support management over the study period, a sensitivity analysis was performed after the exclusion of patients who received a PC-ECMO before 2011.

## Results

**Figure 1:**
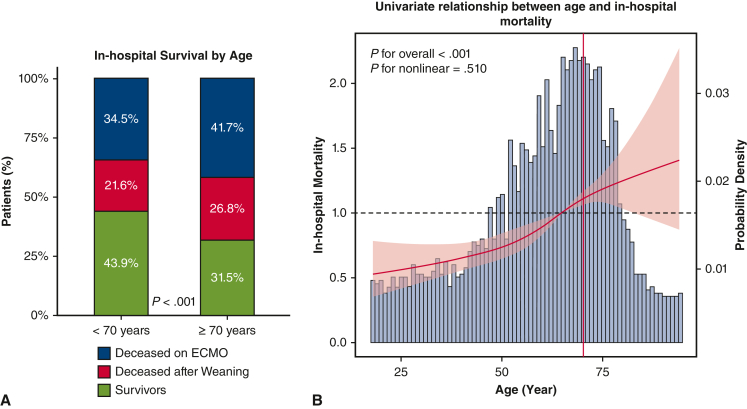
A, Bar chart representing in-hospital survival stratified by age (<70 years old vs ≥70 years old). B, Relationship between age and in-hospital mortality by restricted cubic spline plot. Knots were located at age values of 35, 59, 69, 79 years, corresponding to the 5th, 35th, 65th, and 95th percentiles. The *vertical red line* indicates the age of 70 years.

The study included 2057 patients (Online Data Supplement), of whom 1376 patients (67%) were in the <70 year group with a median age of 59 years (first and third quartile, 18-69.9), and 680 patients (33%) were in the ≥70 years group with a median age of 75 years (first and third quartile, 70-94 years) (Figure 1, *A*). The ≥70 years group was characterized by a greater prevalence of hypertension, atrial fibrillation, diabetes mellitus, peripheral arterial disease, pulmonary hypertension, and valve and coronary diseases (Table 1). The <70 years group had a worse preoperative hemodynamic status with a lower left ventricular ejection fraction (*P* < .001), more frequent preoperative intubation (*P* = .009) and vasopressors use (*P* = .004), and experienced more from aortic disease (*P* = .011) and endocarditis (*P* = .048). They underwent more aortic procedures (*P* = .002) and had a longer cardiopulmonary bypass (CPB) time (*P* = .001; Table 2). Median ECMO duration was 4.9 days (first and third quartile: 2.4-8.0) with 62.5% (n = 1286) initiated intraoperatively (Online Data Supplement) and no differences in both groups.

**Table 1:** Demographics and preoperative characteristics of the overall population

Variable	Overall population (n = 2056)	Age group		*P* value
		<70 y (n = 1376)	≥70 y (n = 680)	
Age, y	65 (55-72)	59 (51-65)	74.1 (72-77.9)	<.001
Sex				.166
Female	843 (41)	594 (39.3)	293 (43)	
Male	1213 (59)	827 (60.1)	387 (56.9)	.166
Body mass index, kg/m^2^	26.4 (23.6-30)	26.5 (23.4-30)	26.2 (23.8-29.7)	.887
Hypertension	1357 (66)	779 (58.7)	531 (80.8)	<.001
Dialysis	183 (8.9)	130 (9.7)	48 (7.4)	.084
Myocardial infarction (last 30 d)	241 (11.7)	137 (10.3)	95 (14.5)	.007
Smoking	553 (26.9)	354 (30)	116 (20.5)	<.001
Atrial fibrillation	541 (26.3)	309 (22.4)	231 (34)	<.001
Previous stroke	284 (13.8)	184 (13.4)	100 (14.7)	.406
Diabetes mellitus	520 (25.3)	311 (22.6)	210 (30.9)	<.001
COPD	214 (10.4)	124 (9.4)	82 (12.6)	.026
Peripheral artery disease	302 (14.7)	173 (12.6)	129 (19)	<.001
Pulmonary hypertension (>50 mm Hg)	430 (20.9)	258 (18.9)	170 (25.1)	.001
Previous cardiac surgery	541 (26.3)	392 (28.5)	149 (21.9)	.001
Left ventricular ejection fraction, %	45 (30-60)	45 (27-60)	50 (35-60)	<.001
Preoperative creatinine, μmol/L	101.6 (79.5-140.5)	100 (79.5-137.9)	105.6 (83.1-141.4)	.055
New York Heart Association functional class				.005
Class I	97 (4.7)	104 (8.1)	40 (6)	
Class II	442 (21.5)	280 (21.7)	140 (21.1)	
Class III	808 (39.3)	473 (36.7)	295 (44.5)	
Class IV	654 (31.8)	433 (33.6)	188 (28.4)	
Preoperative condition				
Cardiogenic shock	440 (21.4)	294 (21.6)	140 (21)	.734
Intubation	232 (11.3)	173 (12.6)	59 (8.7)	.009
Urgent surgery	454 (22.1)	319 (23.4)	131 (19.5)	.050
Emergency surgery	535 (26)	351 (25.8)	177 (26.4)	.762
Cardiac arrest	191 (9.3)	120 (8.8)	69 (10.3)	.278
Vasopressors	317 (15.4)	233 (17.1)	82 (12.1)	.004
Right ventricular failure	206 (10)	126 (10.4)	55 (9.3)	.487
Preoperative diagnosis				
Coronary artery disease	991 (48.2)	583 (42.3)	408 (60)	<.001
Aortic vessel disease	335 (16.3)	245 (17.8)	91 (13.4)	.011
Aortic valve disease	701 (34.1)	418 (30.4)	283 (41.6)	<.001
Mitral valve disease	701 (34.1)	431 (31.3)	271 (39.9)	<.001
Tricuspid valve disease	329 (16)	197 (14.3)	133 (19.6)	.002
Post-AMI ventricular septal rupture	58 (2.8)	36 (2.6)	22 (3.2)	.424
Free wall/papillary muscle rupture	37 (1.8)	30 (2.2)	8 (1.2)	.112
Active endocarditis	148 (7.2)	110 (8)	38 (5.6)	.048

Data are reported as n (% as valid percentage, excluding missing values) or median (first and third quartile). *COPD*, Chronic obstructive pulmonary disease; *AMI*, acute myocardial infarction.

**Table 2:** Procedural and extracorporeal membrane oxygenation (ECMO) characteristics

Variable	Overall population (n = 2056)	Age group		*P* value
		<70 y (n = 1376)	≥70 y (n = 680)	
Weight of surgery				<.001
Unknown	13 (0.6)	13 (0.9)	0 (-)	
Isolated CABG	369 (17.9)	225 (16.3)	144 (21.2)	
Isolated non-CABG	1152 (56)	827 (60.1)	325 (47.8)	
Two procedures	148 (7.2)	79 (5.7)	69 (10.1)	
Three or more procedures	375 (18.2)	233 (16.9)	142 (20.9)	
CABG	911 (44.3)	538 (39.1)	373 (54.9)	<.001
Aortic valve surgery	714 (34.7)	434 (31.5)	280 (41.2)	<.001
Mitral valve surgery	647 (31.5)	401 (29.1)	246 (36.2)	.001
Tricuspid valve surgery	275 (13.4)	164 (11.9)	111 (16.3)	.006
Aortic surgery	382 (18.6)	281 (20.4)	101 (14.9)	.002
Aortic surgery type				.690
Aortic root	55 (14.6)	39 (14.0)	16 (16.0)	
Ascending aorta and root	112 (29.6)	84 (30.2)	28 (28.0)	
Ascending aorta	102 (27)	71 (25.5)	31 (31.0)	
Ascending aorta and arch	88 (23.3)	69 (24.8)	19 (19.0)	
Aortic arch/descending aorta	21 (5.6)	15 (5.4)	6 (6.0)	
LVAD	23 (1.1)	20 (1.5)	3 (0.4)	.04
Heart transplantation	209 (10.2)	205 (14.9)	4 (0.6)	<.001
Crossclamp time, min	99 (64-148)	100 (65-152)	95 (63-141.5)	.406
CPB time, min	204 (139.5-288)	210 (145-296)	189 (130-270)	.001
IABP implantation during hospital admission	620 (30.5)	420 (30.9)	200 (29.7)	.577
Preoperative	192 (31)	139 (33.1)	53 (26.5)	
Intraoperative	428 (69)	281 (66.9)	147 (73.5)	
Left ventricular unloading	519 (30.8)	364 (32)	155 (28.4)	.137
ECMO indication				.022
Failure to wean	787 (39.2)	522 (39)	265 (39.6)	
Acute pulmonary embolism	3 (0.1)	2 (0.1)	1 (0.1)	
Arrhythmia	43 (2.1)	33 (2.5)	10 (1.5)	
Cardiac arrest	170 (8.5)	115 (8.6)	55 (8.2)	
Cardiogenic shock	506 (25.2)	313 (23.4)	193 (28.8)	
Pulmonary hemorrhage	9 (0.4)	5 (0.4)	4 (0.6)	
Right ventricular failure	240 (11.9)	170 (12.7)	70 (10.4)	
Respiratory failure	72 (3.6)	45 (3.4)	27 (4)	
Biventricular failure	149 (4.7)	107 (8)	42 (6.3)	
Other	30 (1.5)	27 (2)	3 (0.4)	
ECMO implantation timing				.240
Intraoperative	1286 (62.5)	873 (63.4)	413 (60.7)	
Postoperative	771 (37.5)	504 (36.6)	267 (39.3)	
Cannulation approach				<.001
Unknown	45 (2.2)	15 (1.1)	30 (4.4)	
Only central cannulation	341 (16.6)	234 (17.0)	107 (15.7)	
Only peripheral cannulation	965 (46.9)	675 (49.0)	290 (42.6)	
Mixed/switch cannulation	706 (34.3)	453 (32.9)	253 (37.2)	
Open chest	634 (42.5)	403 (40.9)	231 (45.6)	.085
ECMO duration, d	4.9 (2.5-8.0)	5 (2.5-8.5)	4.8 (2.4-7.0)	.139

Data are reported as n (% as valid percentage, excluding missing values) or median (first and third quartile). *CABG*, Coronary artery bypass grafting; *LVAD*, left ventricular assist device; *CPB*, cardiopulmonary bypass; *IABP*, intra-aortic balloon pump; *ECMO*, extracorporeal membrane oxygenation.

**Figure 2:**
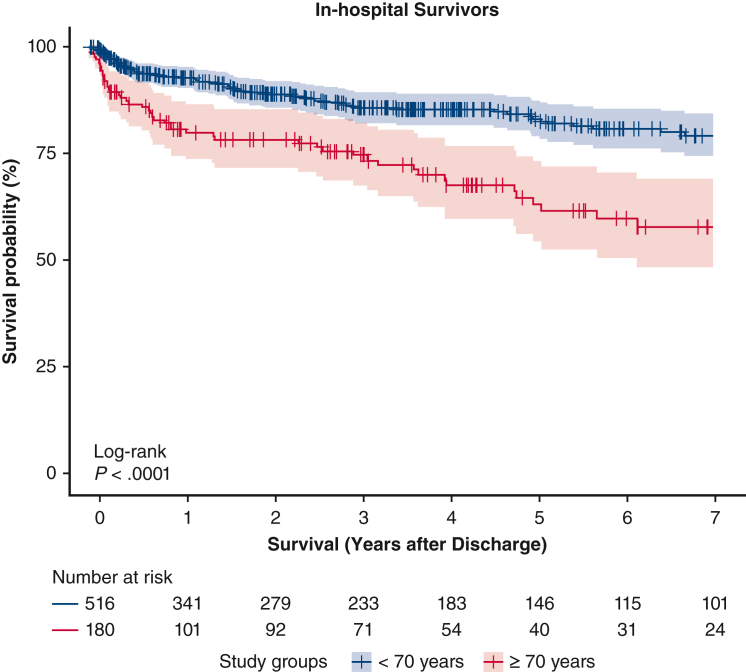
Postdischarge survival for patients who were discharged alive as represented by Kaplan-Meier curve with 95% confidence intervals.

Leg and bowel ischemia occurred more frequently in <70 years patients (*P* = .005 and *P* = .019, respectively; Table 3). The ≥70 years group had a greater in-hospital mortality rate (<70 years: n = 775, 56.3%; ≥70 years: n = 468, 68.8%; *P* < .001) both during ECMO support and after weaning (Figure 1, *A*). Age showed a yearly HR of 1.33 (95% CI, 1.23-1.44) for in-hospital mortality (Figure 1, *B*). The postdischarge survival was greater in patients <70 years when compared with those ≥70 years (*P* < .001, Figure 2). After we excluded patients operated on before 2011, results were confirmed (Online Data Supplement).

**Table 3:** Postoperative complications and mortality

Variable	Overall population, n = 2056	Age group		*P* value
		<70 y (n = 1376)	≥70 y (n = 680)	
Postoperative bleeding	1156 (57.2)	761 (56.4)	395 (58.8)	.311
Requiring rethoracotomy	765 (39.7)	500 (38.7)	265 (41.9)	.183
Cannulation site bleeding	246 (12.2)	160 (11.8)	86 (12.9)	.509
Cerebral hemorrhage	66 (3.4)	45 (3.4)	21 (3.3)	.895
Ischemic stroke	217 (10.6)	153 (11.2)	64 (9.5)	.236
Leg ischemia	200 (10.3)	152 (11.6)	48 (7.5)	.005
Bowel ischemia	107 (5.7)	83 (6.5)	24 (3.9)	.019
Right ventricular failure	389 (21)	249 (19.9)	140 (23.3)	.090
Acute kidney injury	1069 (56.7)	708 (55.8)	361 (58.6)	.255
Septic shock	310 (16.8)	217 (17.4)	93 (15.5)	.320
ARDS	104 (5.5)	71 (5.6)	33 (5.3)	.829
Multiorgan failure	697 (34.3)	443 (32.7)	254 (37.7)	.025
In-hospital mortality	1243 (60.4)	775 (56.3)	468 (68.8)	<.001
Cause of in-hospital mortality				.931
Multiorgan failure	431 (34.6)	271 (37.3)	160 (37)	
Sepsis	85 (6.8)	56 (7.7)	29 (6.7)	
Persistent heart failure	423 (34)	263 (36.2)	160 (37)	
Distributive shock syndrome	22 (1.8)	14 (1.9)	8 (1.9)	
Bleeding	64 (5.1)	43 (5.9)	21 (4.9)	
Neurologic	58 (4.6)	33 (4.5)	25 (5.8)	
Bowel ischemia	22 (1.8)	15 (2.1)	7 (1.6)	
Other	53 (4.3)	31 (4.3)	22 (5.1)	

Data are reported as n (% as valid percentage, excluding missing values) or median (first and third quartile). *ARDS*, Acute respiratory distress syndrome.

### Subgroup Analysis and Variables Associated With Mortality in the ≥70 Years Group

In patients ≥70 years, in-hospital nonsurvivors had greater median preoperative creatinine values (in-hospital survivors: 99 μmol/L, first and third quartile, 79.6-125; in-hospital nonsurvivors: 107 μmol/L, first and third quartile, 85-150; *P* = .011), and a history of previous stroke (in-hospital survivors: n = 22, 10.4%; in-hospital nonsurvivors: n = 78, 16.7%; *P* = .032), experienced more often preoperative cardiogenic shock (in-hospital survivors: n = 31, 15%; in-hospital nonsurvivors: n = 109, 23.6%; *P* = .011) and right ventricular failure (in-hospital survivors: n = 9, 4.9%; in-hospital nonsurvivors: n = 46, 11.3%; *P* = .013; Online Data Supplement), and underwent more aortic surgery (in-hospital survivors: n = 24, 11.3%; in-hospital nonsurvivors: n = 77, 16.5%; *P* = .08) with longer CPB time (in-hospital survivors: 178 minutes, first and third quartile, 120-243; in-hospital nonsurvivors: 199 minutes, first and third quartile, 135-285; *P* = .01; Online Data Supplement). They experienced more postoperative complications (Online Data Supplement), including bleeding (in-hospital survivors: n = 102, 48.8%; in-hospital nonsurvivors: n = 293, 63.3%; *P* < .001), ischemic stroke (in-hospital survivors: n = 30, 14.2%; in-hospital nonsurvivors: n = 34, 7.3%; *P* = .005), bowel ischemia (in-hospital survivors: n = 2, 1%; in-hospital nonsurvivors: n = 22, 5.1%; *P* = .015), and right ventricular failure (in-hospital survivors: n = 30, 16.4%; in-hospital nonsurvivors: n = 110, 26.4%; *P* = .008).

In a mixed-effects Cox model with random center and year effects, age showed a yearly hazard of 1.03 (95% CI, 1.0-1.1, *P* = .036; Online Data Supplement) for in-hospital mortality. Further variables associated with in-hospital mortality included stroke (HR, 1.39; 95% CI, 1.1-1.8), preoperative right ventricular failure (HR, 1.45; 95% CI, 1.0-2.1), and aortic surgery (HR, 1.65; 95% CI, 1.2-2.2). Similarly, postoperative complications associated with greater hazards of in-hospital mortality included bleeding (HR, 1.24; 95% CI, 1.0-1.5), cardiac arrest (HR, 1.65; 95% CI, 1.3-2.1), and right ventricular failure (HR, 1.29; 95% CI, 1.0-1.6).

### Subgroup Analysis of the ≥80 Years Age Group

In total, 91 (4.4%) patients were included in this group (Online Data Supplement). Most variables were similar to the ≥70 years group except for a greater preoperative creatinine (141.1 μmol/L, first and third quartile, 1.1-539.4) and greater incidences of aortic valve disease (n = 51, 56%) and aortic valve surgery (n = 47, 51.6%). The in-hospital mortality rate was 70.3% (n = 64).

## Discussion

This study has 5 main findings. First, ≥70 years patients had more preoperative comorbidities, better preoperative cardiac function, and underwent less-complicated operations including valve and coronary artery bypass grafting procedures compared with the <70 years group. Second, patients <70 years experienced more acute preoperative deterioration (intubation and vasopressors) and underwent more complex operations with a longer CPB time. Third, in-hospital and postdischarge mortality was greater in patients ≥70 years compared with the younger population, with a yearly HR of 1.33 (95% CI, 1.23-1.44) for in-hospital mortality. Fourth, in patients ≥70 years, variables associated with greater in-hospital mortality included preoperative conditions (previous stroke and right ventricular failure), type of operation (aortic surgery), and postoperative complications (bleeding, cardiac arrest, and right ventricular failure). Fifth, patients who were octogenarian were 4.4% of the population with an in-hospital mortality rate of 70.3%.

Older patients are known to be a fragile category in cardiac surgery, and advanced age has been identified as consistently associated with increased in-hospital mortality after PC- ECMO.^3^^,^^6^ For this reason, even if age is not an absolute contraindication for ECMO, clinicians might hesitate to initiate venoarterial ECMO in older patients, especially because they are rarely candidates for heart transplant or durable mechanical circulatory support.^13^ Nevertheless, so far, there are no tools to help in patient selection for ECMO when age is advanced, and this population has been poorly investigated. This study showed that patients ≥70 years underwent more frequent operations for valve or coronary artery diseases, which are age-related conditions, and fewer aortic operations compared with patients <70 years. Moreover, aortic surgery carried a 1.65 (95% CI, 1.24-2.20) times increased risk for in-hospital mortality in patients ≥70 years, suggesting that this kind of operation is a greater-risk procedure associated with worse outcomes when requiring PC-ECMO. Although older age is known to be a risk factor for mortality in aortic surgery, some authors suggested that older age should not be an absolute contraindication on the basis of retrospective analysis.^14^ However, it is known that patients who were octogenarian often refused complicated ascending aortic and aortic arch surgery because of the previously published poor outcomes when compared with younger cohorts.^15^ This might lead to selection bias in which patients who are older die perioperatively, before ECMO support.

Similarly, a major selection bias could explain the lower rate of preoperatively unstable patients requiring intubation and vasopressors in the ≥70 years group compared with the <70 years group and their better cardiac function, less complicated procedures, and shorter CPB time. In case of a patient who is older, fragile, and unstable, surgeons and intensivists tend to discuss alternative therapies with the patient and his or her family. In many cases, there is mutual agreement not to support the patient with ECMO because of the known greater mortality and the potential severe morbidity associated with advanced age. Results of this study suggest that, in clinical practice, older patients probably receive PC-ECMO if their overall conditions are less complex whereas those with expected greater risks of negative outcomes are excluded from advanced circulatory support.

Interestingly, even although younger patients had more unstable preoperative conditions, more complex operations and more complications such as leg and bowel ischemia, they still had a 13% better survival compared with patients ≥70 years (56.3% vs 68.8%). Moreover, 21.6% of the patients <70 years and 26.8% of the patients ≥70 years died after successful ECMO weaning. The explanation for this phenomenon can be multifactorial, and its analysis might identify factors that can be modified to improve survival in older patients. Besides age itself, this study identified preoperative conditions (previous stroke and right ventricular failure), type of operation (aortic surgery), and postoperative complications (bleeding, cardiac arrest, and right ventricular failure) as variables associated with in-hospital mortality in patients ≥70 years. Timing of ECMO implantation as well as preoperative hemodynamic and urgency status are other variables that might affect survival after PC-ECMO.^16^ It was previously demonstrated that intraoperative and postoperative ECMO implantations are associated with different patient characteristics and outcomes, with greater complications and in-hospital mortality after postoperative ECMO.^17^ All modifiable factors identified by this study could be a possible target for care improvement in older patients. Moreover, they might contribute to a better preoperative risk stratification to direct the patient toward a less-invasive treatment or to better coordinate expectations with the families pre and postoperatively.

Age itself is associated with increased mortality in cardiogenic shock^18^ and PC-ECMO studies.^6^^,^^19^ This controversy might be a sign that age itself is not the problem, but the associated conditions might be. In fact, most preoperative diseases are age-related, and many of them are linked to greater postoperative mortality. Other independent variables for mortality such as bleeding, renal-replacement therapy, lactate level, and others were described in different studies.^6^^,^^18^, ^19^, ^20^, ^21^ Right ventricular failure has also been associated with mortality in patients who undergo ECMO postoperatively. Bartko and colleagues^22^ showed that echocardiographic signs of right ventricular dysfunction postcardiac surgery were strongly associated with a negative impact on survival in patients supported by ECMO postoperatively. Finally, almost all postoperative ECMO complications that were analyzed were identified as independent variables for increased mortality in the older age group.

These findings are in line with other publications that found an increased overall mortality rate in older age populations in-hospital, and postdischarge.^19^ In contrast, approximately one quarter of patients ≥70 years receiving PC-ECMO are alive 2 years after surgery, indicating that age >70 years is not an absolute contraindication to PC-ECMO. The percentage of patients who are older and who are fragile undergoing cardiac surgery has increased over the past 15 years^3^; therefore, although it may seem low, it still represents a significant number of patients. Further studies are required to investigate the postdischarge quality of life after cardiac surgery and ECMO in older patients, as well as the caregiver burden in case of permanent disability.^23^^,^^24^

### Strengths and Limitations

This is a retrospective observational study, which prevents causal inferences and suffers from confounding by indication and selection bias, as previously discussed. Analyzing 20 years of patients undergoing PC-ECMO in 34 centers provides a wide view of the field and powered analysis. However, over the years ECMO care and technology have evolved, which may create confounding. We performed a sensitivity analysis in an attempt to overcome these factors. Participation in the PELS registry was on a voluntary basis. We presume that some centers did not provide data on all eligible patients consecutively. A partial overlapping with previously reported series cannot be excluded.^25^ The database did not capture the diagnosis of the acute aortic syndrome leading to emergency aortic surgery. Thus, it was not possible to identify patients with type A acute aortic dissection. Only selected perioperative data were collected, so not all ECMO-related variables or details of the patients’ hemodynamic status are available. Selection bias and confounding by indication can be postulated in the older population. Clinicians are more liberal in supporting younger patients with ECMO postcardiotomy and tend to limit the extent of the treatment in older patients selecting only those in better conditions, thus leading to an underestimation of negative results. Studies are required to further investigate the effect of this selection bias.

## Conclusions

Older age is associated with high mortality in patients who receive PC-ECMO. However, knowing that other perioperative variables are also involved, better patient selection and postoperative treatment might improve survival. Patients who are elderly with a preoperative history of stroke and right ventricular failure, or undergoing aortic surgery, should be carefully evaluated during the selection process for PC- ECMO, on the basis of their greater risks of negative outcomes. Efforts are required to prevent postoperative complications such as postoperative bleeding, stroke, cardiac arrest, and right ventricular failure, which are associated with increased mortality in elderly patients.

### Webcast

You can watch a Webcast of this AATS meeting presentation by going to: Xxx.

## Conflict of Interest Statement

R.L. reported consultant for Medtronic, Getinge, Abiomed, and LivaNova and advisory board member of Eurosets, Hemocue, and Xenios (honoraria as research funding). D.W. reported consultant/proctor for Abbott and scientific advisor for Xenios. G.M. is the Past President of The Extracorporeal Life Support Organization (ELSO). All other authors reported no conflicts of interest.

The *Journal* policy requires editors and reviewers to disclose conflicts of interest and to decline handling or reviewing manuscripts for which they may have a conflict of interest. The editors and reviewers of this article have no conflicts of interest.
