# Supplementary material for: Postcardiotomy extracorporeal membrane oxygenation in patients older than 70 years: Characteristics, outcomes, and variables associated with mortality
Source: JTCVS Open. 2025 May 3;25:163–72. doi: 10.1016/j.xjon.2025.04.004 (PMC12230581; doi:10.1016/j.xjon.2025.04.004)
Supplement: Online Data Supplement [file mmc1.docx]

***Electronic Supplementary Material***

**Post-cardiotomy Extracorporeal Membrane Oxygenation in Patients Older than 70 Years: characteristics, outcomes, and variables associated with mortality**

**Corresponding author:**Maged Makhoul,

Cardiovascular Research Institute Maastricht,

University of Maastricht,

P. Debyelaan, 25

6202AZ, Maastricht, The Netherlands

Email: magedmakhoul@gmail.com

Complete affiliations and list of PELS Investigators

Maged Makhoul^1,2^, Silvia Mariani^3,4^, Bas C.T. van Bussel^4,5,6^, Samuel Heuts^1^, Michele Di Mauro^1^, Dominik Wiedeman^7,8^, Ann-Kristin Schaefer^7^, Luca Conci^7^, Diyar Saeed^9^, Jawad Khalil^9^, Sven Lehmann^9^, Matteo Pozzi^10^, Jean-Francois Obadia^10^, Antonio Loforte^11,12^, Luca Botta^11^, Davide Pacini^11^, Udo Boeken^13^, Nikolaos Kalampokas^13^, Robertas Samalavicius^14^, Agne Jankuviene^14^, Karl Bounader^15^, Erwan Flecher^15^, Xiaotong Hou^16^, Jeroen J.H. Bunge^17,18^, Dinis Dos Reis Miranda^18^, Hergen Buscher^19^, Kogulan Sriranjan^19^, Leonardo Salazar^20^, Bart Meyns^21^, Daniel Herr^22^, Michael A Mazzeffi^22^, Sacha Matteucci^23^, Marco Di Eusanio^23^, Sandro Sponga^24^, Igor Vendramin^24^, Graeme MacLaren^25^, Vitaly Sorokin^25^, Kollengode Ramanathan^25^, Claudio Russo^26^, Alessandro Costetti^26^, Francesco Formica^3,27^, Giovanni Marchetto^3^, Pranya Sakiyalak^28^, Antonio Fiore^29^, Daniele Camboni^30^, Chistof Schmid^30^, Giuseppe Maria Raffa^31,32^, Rodrigo Diaz^33^, Roberto Castillo^33^, I-wen Wang^34^, Jae-Seung Jung^35^, Jan Belohlavek^36^, Tomas Grus^37^, Vin Pellegrino^38^, Giacomo Bianchi^39^, Marco Solinas^39^, Matteo Pettinari^40^, Alessandro Barbone^41^, José P. Garcia^42^, Kiran Shekar^43^, Glenn Whitman^44^, Gil Bolotin^2^, Roberto Lorusso^1^.

1. Cardio-Thoracic Surgery Department, and Cardiovascular Research Institute Maastricht, Maastricht, The Netherlands.
2. Cardiac Surgery Department, Rambam Medical centre. Haifa, Israel.
3. Cardiac Surgery Unit, Cardio-thoracic and Vascular Department, Fondazione IRCCS San Gerardo dei Tintori, Monza, Italy.
4. Cardiovascular Research Institute Maastricht (CARIM), Maastricht, The Netherlands.
5. Department of Intensive Care Medicine, and Cardiovascular Research Institute Maastricht, Maastricht, The Netherlands.
6. Care and Public Health Research Institute, Maastricht University, Maastricht, The Netherlands.
7. Department of Cardiac Surgery, Medical University of Vienna, Vienna, Austria.
8. Department of Cardiac Surgery, Karl Landsteiner University, University Clinic St, Pölten, St. Pölten, Austria.
9. Department of Cardiac Surgery, Leipzig Heart Center, Leipzig, Germany
10. Department of Cardiac Surgery, Louis Pradel Cardiologic Hospital, Lyon, France
11. Division of Cardiac Surgery, IRCCS Azienda Ospedaliero-Universitaria di Bologna, Bologna, Italy.
12. University of Turin, Turin, Italy
13. Department of Cardiac Surgery, Medical Faculty, Heinrich Heine University, Duesseldorf, Germany.
14. II Department of Anesthesiology, Centre of Anesthesia, Intensive Care and Pain management, Vilnius University Hospital Santariskiu Klinikos, Vilnius, Lithuania.
15. Division of Cardiothoracic and Vascular Surgery, Pontchaillou University Hospital, Rennes, France.
16. Center for Cardiac Intensive Care, Beijing Institute of Heart, Lung, and Blood Vessels Diseases, Beijing Anzhen Hospital, Capital Medical University, Beijing, China.
17. Department of cardiology, Erasmus MC, Rotterdam, The Netherlands.
18. Department of Intensive Care Adults, Erasmus MC, Rotterdam, The Netherlands.
19. Department of Intensive Care Medicine, Center of Applied Medical Research, St Vincent's Hospital, Darlinghurst, NSW, Australia.
20. Department of Cardiology, Fundación Cardiovascular de Colombia, Bucaramanga, Colombia.
21. Department of Cardiac Surgery, University Hospitals Leuven and Department of Cardiovascular Sciences, University of Leuven, Leuven, Belgium.
22. Departments of Medicine and Surgery, University of Maryland, Baltimore, USA.
23. SOD Cardiochirurgia Ospedali Riuniti 'Umberto I - Lancisi - Salesi' Università Politecnica delle Marche, Ancona, Italy.
24. Division of Cardiac Surgery, Cardiothoracic Department, University Hospital of Udine, Udine, Italy.
25. Cardiothoracic Intensive Care Unit, National University Heart Centre, National University Hospital, Singapore, Singapore.
26. Cardiac Surgery Unit, Cardiac Thoracic and Vascular Department, Niguarda Hospital, Milan, Italy.
27. Department of Medicine and Surgery, University of Parma, Parma, Italy.
28. Division of Cardiovascular and Thoracic Surgery, Department of Surgery, Faculty of Medicine Siriraj Hospital, Mahidol University, Bangkok, Thailand.
29. Department of Cardio-Thoracic Surgery, University Hospital Henri-Mondor, Créteil, Paris, France.
30. Department of Cardiothoracic Surgery, University Medical Center Regensburg, Regensburg, Germany.
31. Cardiac Surgery Unit, Department of Precision Medicine in Medical Surgical and Critical Area (Me.Pre.C.C.), University of Palermo, 90134 Palermo, Italy.
32. Department for the Treatment and Study of Cardiothoracic Diseases and Cardiothoracic Transplantation, IRCCS-ISMETT (Istituto Mediterraneo per i Trapianti e Terapie ad Alta Specializzazione), Palermo, Italy.
33. ECMO Unit, Departamento de Anestesia, Clínica Las Condes, Las Condes, Santiago, Chile.
34. Division of Cardiac Surgery, Memorial Healthcare System, Hollywood, FL 33021, USA.
35. Department of Thoracic and Cardiovascular Surgery, Korea University Anam Hospital, Seoul, South Korea.
36. 2nd Department of Internal Medicine, Cardiovascular Medicine General Teaching Hospital and 1st Faculty of Medicine, Charles University in Prague, Prague, Czech Republic.
37. 2nd Department of Cardiovascular Surgery, First Faculty of Medicine, Charles University and General University Hospital in Prague, Prague, Czech Republic.
38. Intensive Care Unit, The Alfred Hospital, Melbourne, VIC, Australia.
39. Ospedale del Cuore Fondazione Toscana "G. Monasterio", Massa, Italy.
40. Department of Cardiovascular Surgery, Ziekenhuis Oost-Limburg, Genk, Belgium.
41. Cardiac Surgery Unit, IRCCS Humanitas Research Hospital – Rozzano (MI) – Italy.
42. IU Health Advanced Heart & Lung Care, Indiana University Methodist Hospital, Indianapolis, IN, USA.
43. Adult Intensive Care Services, The Prince Charles Hospital, Brisbane, Australia.
44. Cardiac Intensive Care Unit, Johns Hopkins Hospital, Baltimore, Maryland, USA.

Supplemental Methods

**Data collection**

The following predefined groups of data were collected:

- Demographic data: sex, age, race
- Patients characteristics: EuroSCORE, length, weight, serum creatinine level, left ventricular ejection fraction, comorbidities (hypertension, chronic kidney disease requiring dialysis, previous myocardial infarction, previous endocarditis, smoking, previous stroke, atrial fibrillation, previous pulmonary embolism, diabetes mellitus, previous transient ischemic attack (TIA), implanted pacemaker (PM), implanted implantable cardioverter defibrillator (ICD), previous percutaneous coronary intervention (PCI), chronic obstructive pulmonary disease (COPD), peripheral artery disease, chronic pulmonary embolism, asthma, pulmonary hypertension, previous cardiac surgery, implanted left ventricular assist device (LVAD), New-York Heart Association class.
- Preoperative status: urgency of the procedure, weight of intervention, planned intervention, preoperative cardiogenic shock, preoperative intubation, preoperative cardiac arrest, preoperative septic shock, preoperative vasopressors, preoperative acute pulmonary oedema, preoperative intra-aortic balloon pump (IABP), preoperative right ventricular failure, preoperative biventricular failure.
- Diagnosis: coronary artery disease, aortic vessel disease, aortic valve disease, mitral valve disease, tricuspid valve disease, pulmonary valve disease, post-acute myocardial infarction (AMI) ventricular septal rupture, free wall/Papillary muscle rupture, graft failure, active endocarditis, atrial septal defect, post-LVAD right ventricular failure, other diagnosis
- Coronary surgery: arterial graft, number of distal arterial anastomoses, left internal mammary artery (LIMA), right internal mammary artery (RIMA), radial artery, gastro-epiploic artery (GEA), other arterial graft, venous graft, number of distal venous anastomoses, other coronary surgery
- Valve surgery: valve surgery, aortic valve surgery, aortic valve procedure, mitral valve surgery, mitral valve procedure, pulmonary valve surgery, pulmonary valve procedure, pulmonary valve implant, tricuspid valve surgery, tricuspid valve procedure.
- Aortic surgery: approach to aortic surgery, aortic ascending surgery, aortic arch surgery, descending aortic procedure.
- Other cardiac surgeries: cardiac assist device, heart transplantation, rhythm surgery, additional PM-/ICD procedure, ventricular septal defect (VSD) closure, atrial septal defect (ASD) closure, ventricular surgery, pericardiectomy, pulmonary embolectomy/endarterectomy, other cardiac surgery, other cardiac surgery description.
- Extracorporeal circulation (ECC): ECC duration, cross-clamp duration, circulation arrest, cardioplegia characteristics, off-pump conversion.
- Extracorporeal membrane oxygenation (ECMO) variables: ECMO indication, chest status, cannulation approach, use of left ventricular vent, ECMO duration (hours), configuration change, ECMO monitoring.
- In-hospital outcomes: deceased in hospital, deceased timing, intensive care unit stay (days), hospital stay (days), in-hospital mortality, death timing, postoperative bleeding (requiring re-thoracotomy, cannulation site bleeding, diffuse no-surgical related bleeding), neurological complications (brain oedema, cerebral haemorrhage, seizure, stroke, vasospasm), arrhythmia, leg ischemia, cardiac arrest, pacemaker implant, bowel ischemia, right ventricular failure, acute kidney injury, pneumonia, septic shock, distributive shock syndrome, acute respiratory distress syndrome (ARDS), multi-organ failure, embolism
- Postoperative procedures: PCI, new cardiac surgery, abdominal surgery, vascular surgery
- Outcomes at follow-up: mortality status, follow-up time

**Variable definitions**

The following definitions were used for the main study variables:

- Sex: our research and manuscript have been developed in accordance to the international World Health Organization definitions of sex and gender where ‘Gender’ describes those characteristics of women and men that are largely socially created (including concepts such as cisgender and transgender). while ‘sex’ encompasses those that are biologically determined (https://www.who.int/genomics/gender/en/). The same distinction is mirrored by the definitions of gender and sex given by several other international institutions such as the World Health Organization Regional Office for Europe (https://www.euro.who.int/en/health-topics/health-determinants/gender/gender-definitions), the Office for National Statistics and United Kingdom government (https://www.ons.gov.uk/economy/environmentalaccounts/articles/whatisthedifferencebetweensexandgender/2019-02-21) or the Canadian Institutes of Health Research (https://cihr-irsc.gc.ca/e/48642.html). Despite these definitions, sex and gender are often mistakenly used interchangeably in scientific literature, health policy, and legislation. In our study, we defined our patients based on biologically determined sex (male/female) and we did not include a further analysis of gender identity ^1-3^.
- Hypertension: Systolic blood pressure >140mmHg or diastolic blood pressure >90mmHg^4^, or use of antihypertensive agents to maintain normal blood pressure
- Smoking: active (smoking during the past 30 days) and more than 100 cigarettes during lifetime
- COPD: Diagnosis of chronic obstructive pulmonary disease, any Gold classification ^5^
- Peripheral arterial disease: Claudication, carotid occlusion or >50% stenosis, amputation for arterial disease or previous or planned intervention on the abdominal aorta. limb arteries or carotids ^6^
- Dialysis: chronic kidney disease requiring dialysis before hospital admission for the indexed procedure.
- Pulmonary hypertension: Systolic pulmonary artery pressure >50mmHg
- EuroSCORE II: European System for Cardiac Operative Risk Evaluation II proposing a risk assessment of cardiac surgical procedures which incorporates patient age, sex, diabetic status, pulmonary disease, neurological function, renal function, presence of active endocarditis, pre-operative state, procedural urgency and procedure type^6^
- New York heart association class: Functional class of dyspnoea according to the classification as proposed by the New York Heart Association
- Preoperative cardiogenic shock: Preoperative state with life-threatening hypotension despite rapidly escalating inotropic support. critical organ hypoperfusion, with worsening acidosis and/or lactate levels^7^
- Preoperative cardiac arrest: Preoperative cardiopulmonary resuscitation in the 24 hours prior to surgery
- Preoperative septic shock: Septic patients with vasopressor requirement to maintain mean arterial pressure >65mmHg and serum lactate levels greater than 2mmol/L in the absence of hypovolemia^8^
- Preoperative right ventricular failure: Evidence of right-sided structural and/or functional abnormalities in combination with clinical symptoms and signs of right ventricular failure^9^
- Preoperative biventricular failure: Biventricular dysfunction accompanied by both signs and symptoms of right-sided and left-sided heart failure^10^
- Emergency surgery: Surgery before the beginning of the next working day after the decision to operate is made^6^
- Urgent surgery: Patients not electively admitted for operation but requiring surgery during the current admission without a possibility to be discharged before undergoing the definite procedure ^6^
- Aortic vessel disease: Any disease of the ascending aorta. aortic arch or proximal descending aorta warranting surgical correction during the current procedure
- Aortic valve disease: Any aortic valve disease, including (prosthetic) aortic valve stenosis, regurgitation and endocarditis
- Mitral valve disease: Any mitral valve disease, including (prosthetic) mitral valve stenosis, regurgitation and endocarditis
- Tricuspid valve disease: Any tricuspid valve disease, including (prosthetic) tricuspid valve stenosis. regurgitation and endocarditis
- Pulmonary valve disease: Any pulmonary valve disease, including (prosthetic) pulmonary valve stenosis, regurgitation and endocarditis
- Active endocarditis: Patients still on antibiotic treatment for endocarditis at the time of surgery^6^
- Post LVAD right ventricular failure: Right ventricular failure as described previously in presence of LVAD
- Ventricular surgery: Surgery performed to restore structural ventricular function, especially in case of ventricular aneurysm formation or rupture
- Rhythm surgery: Surgical (either epicardial or endo-epicardial) ablation performed for atrial or ventricular arrhythmia
- Failure to wean: Failure to wean from cardio-pulmonary bypass despite preload optimization and completeness of surgery
- Arrhythmia: Refractory ventricular arrhythmia with uncontrollable hemodynamic consequences
- Cardiac arrest: Abrupt loss of heart function despite acute and simple interventions such as pacing and defibrillation
- Cardiogenic shock: State of life-threatening hypotension despite rapidly escalating inotropic support, critical organ hypoperfusion, with worsening acidosis and/or lactate levels ^7^
- Right ventricular failure: Evidence of right-sided structural and/or functional abnormalities in combination with clinical symptoms and signs of right ventricular failure ^9^
- Respiratory failure: Reversible pulmonary disease which cannot anymore be managed by conventional mechanical ventilation, despite optimization of pharmacological interventions with or without prone positioning
- Biventricular failure: Biventricular dysfunction accompanied by both signs and symptoms of right-sided and left-sided heart failure ^10^
- Chest closed: Any cannulation condition in which the sternum is closed irrespective location of cannulas
- Chest open: Any cannulation condition in which the sternum is left open irrespective of skin closure
- Stroke: Neurological dysfunction caused by focal brain or retinal ischemia with clinical symptoms lasting less more than 24 hours, with or without permanent disability
- Transient ischemic attack: A brief episode of neurological dysfunction caused by focal brain or retinal ischemia with clinical symptoms lasting less than one hour, without evidence of acute brain infarction ^11^
- Arrhythmia: Any atrial or ventricular arrhythmia lasting more than 30 seconds
- Leg ischemia: Clinical signs of lower extremity ischemia requiring intervention (either by vascular surgery or cannula removal)
- Bowel ischemia: Intestinal ischemia with elevated lactate levels requiring abdominal surgical intervention
- Acute kidney injury: Postoperative requirement for dialysis while not on dialysis before or duplication of preoperative creatinine levels (and absolute creatinine level >177μmol/L)
- Pneumonia: Any (suspected) pulmonary infection treated with antibiotics
- Septic shock: Sepsis with vasopressor requirement to maintain mean arterial pressure >65mmHg and serum lactate levels greater than 2mmol/L in the absence of hypovolemia^8^
- Distributive shock syndrome: mean arterial pressure <50mmHg with cardiac index >2,5L/min/m^2^, right atrial pressure <5mmHg, left atrial pressure <10mmHg an low systemic vascular resistance (<800 dyne/s/cm^-5^) during intravenous norepinephrine infusion (>0,5μg/kg/min)^12^
- ARDS: Acute diffuse inflammatory lung injury requiring invasive mechanical ventilation of extracorporeal membrane oxygenation
- Multi-organ failure: Hypometabolic state with involvement of more than one organ as established by biochemical and/or radiological analysis
- ECMO timing: Time of ECMO implantation; intra-operative or post-operative.

**Statistical analysis**

Data were merged and analysed using SPSS 26.0 (IBM. New York. USA), and R 4.1.2 (R Foundation for Statistical Computing. Vienna. Austria). The full cohort was categorized into two study groups (<70y, ≥70y) for comparison. Missing data analysis was conducted with the *mice: Multivariate Imputation by Chained Equations* R package. The percentage of missing values was calculated for each variable (Supplementary Table 1). Missing data patterns were investigated and were identified as missing completely at random (MCAR).

Descriptive statistics were conducted on available data only and no imputations were performed for this purpose. Normality was investigated with Kolmogorov-Smirnov, Shapiro-Wilk, and inspection of histograms and Q-Q plots as appropriate. Homogeneity of variance was tested with the Levene’s test. Demographic and clinical variables were expressed as numbers (percent) for categorical variables. For each 2x2 table, chi-square test was used if all expected cell counts were ≥ 5, otherwise Fisher’s exact test was used. Fisher-Freeman-Halton Exact Test for tables with more than 2x2 cells were used. Continuous variables that complied with normality were reported as means (SD). If equal variances could be assumed, they were tested by independent Student’s T test, otherwise the Welch’s test was used. If they not complied with normality, they were reported as median (1^st^ and 3^rd^ quartile) and tested with a Mann-Whitney U test. To test the association between age and in-hospital mortality, a univariate Cox regression restricted cubic spline model was developed using the rms R package with age as a continuous covariate.

Survival was investigated with the Kaplan–Meier method and comparisons were performed with the Log-rank test (*survival* and *survminer* R packages). Patients’ loss to follow-up were included in survival analyses and were considered censored at the time of their last control. Curves were truncated when the number of patients at risk from the study groups dropped below 10% of the initial sample.

A subgroup analysis was conducted to investigate characteristics and outcomes comparing in-hospital survivors and in-hospital non-survivors among ≥70y patients. To estimate the associations between variables and in-hospital mortality in patients aged ≥ 70 years old, we conducted a mixed-effects Cox proportional hazards regression, using the *Coxme: Mixed Effects Cox Models* R package. The random effect was used to account for the dependency of observations due to clustering in centres and years^13,14^. Based on clinical practice and literature, we first estimated a crude model, which was subsequently adjusted for sets of variables deemed potential confounders for the association with mortality. The mixed-effects Cox proportional hazards and logistic regression models were developed on five imputed datasets after the imputation of variables with <20% missing data. Variables with more missing data were omitted from the models. We used the *mice: Multivariate Imputation by Chained Equations* R package for the imputation process. Five imputed datasets were created with “cart” method. Predictors included variables selected to be part of the mixed-Cox models which were run on each of these datasets, and results were pooled (*junkka/ehahelper: Helper Functions for Event History Analysis* R package) to obtain estimates as hazard ratios (HRs) with their 95% confidence intervals (CIs) and p-values.

A further subgroup analysis was conducted on patients older than 80 years. Based on the possible variations in ECLS management over the study period. A sensitivity analysis was performed after the exclusion of patients who received a post-cardiotomy ECLS before 2011. A two-sided p-value of < 0.05 was considered statistically significant.

**Supplemental Tables**

**Table S1-** Missing cases for each variable.

| **Variable** | **Missing n (%)** |
| --- | --- |
|  |  |
| Age | 0 (0) |
| Sex | 1 (0.05%) |
| Body mass index (kg/m2) | 12 (0.5%) |
| Hypertension | 72 (3.5%) |
| Dialysis | 67 (3.3%) |
| Myocardial infarction (last 30 days) | 71 (3.5%) |
| Smoking | 311 (15.1%) |
| Atrial fibrillation | 1 (0.05%) |
| Stroke | 0 (0) |
| Diabetes mellitus | 0 (0) |
| COPD | 84 (4%) |
| Peripheral artery disease | 0 (0) |
| Pulmonary hypertension (>50mmHg) | 14 (0.6%) |
| Previous cardiac surgery | 0 (0) |
| LVEF | 94 (4.5%) |
| Creatinine level | 147 (7.1%) |
| New York heart association class | 104 (5.1%) |
| Cardiogenic shock | 29 (1.4%) |
| Intubation | 1 (0.05%) |
| Urgent surgery | 20 (0.9%) |
| Emergency surgery | 23 (1.1%) |
| Cardiac arrest | 22 (1.07%) |
| Vasopressors | 17 (0.8%) |
| Right ventricular failure | 255 (12.3%) |
| Coronary artery disease | 0 (0) |
| Aortic vessel disease | 0 (0) |
| Aortic valve disease | 0 (0) |
| Mitral valve disease | 0 (0) |
| Tricuspid valve disease | 0 (0) |
| Post-AMI ventricular septal rupture | 0 (0) |
| Free wall papillary muscle rupture | 0 (0) |
| Active endocarditis | 0 (0) |
| CABG | 0 (0) |
| Aortic valve surgery | 0 (0) |
| Mitral valve surgery | 1 (0.05%) |
| Tricuspid valve surgery | 0 (0) |
| Aortic surgery | 0 (0) |
| LVAD | 0 (0) |
| Heart transplantation | 0 (0) |
| Cross-clamp time | 198 (9.6%) |
| CPB time | 185 (9%) |
| IABP | 23 (1.1%) |
| Left ventricular unloading | 372 (18%) |
| Cannulation approach  ECMO indication | 0 (0)  48 (2.3%) |
| ECMO implant timing | 0 (0) |
| ECMO duration | 37 (1.8%) |
| Chest status | 565 (27.5%) |
| Post-operative bleeding: | 36 (1.7%) |
| Requiring re-thoracotomy | 132 (6.4%) |
| Cannulation site bleeding | 36 (1.7%) |
| Cerebral hemorrhage | 128 (6.2%) |
| Ischemic stroke | 79 (3.8%) |
| Leg ischemia | 111 (5.4%) |
| Bowel ischemia | 166 (8%) |
| Right ventricular failure | 206 (10%) |
| Acute kidney injury | 173 (8.4%) |
| Septic shock | 209 (10.1%) |
| ARDS | 167 (8.1%) |
| Multi-organ failure | 27 (1.3%) |
| In hospital mortality | 0 (0) |

| **Table S2** - Follow-up data for patients grouped based on their age and in-hospital mortality status | | | | | | | | |
| --- | --- | --- | --- | --- | --- | --- | --- | --- |
|  | **Age Groups** | | | | | | | |
|  | **< 70 years (n=1376)** | | | | **≥ 70 years (n=680)** | | | |
|  | **In-hospital Mortality** | | | | **In-hospital Mortality** | | | |
|  | **No (n=602)** | | **Yes (n=775)** | | **No (n=212)** | | **Yes (n=468)** | |
|  | **Median  (1s-3rd quartile)** | **Available cases  N (%)** | **Median  (1s-3rd quartile)** | **Available cases  N (%)** | **Median  (1s-3rd quartile)** | **Available cases  N (%)** | **Median  (1s-3rd quartile)** | **Available cases  N (%)** |
| Hospital stay (days) | 38 (25-60) | 563 (93.5%) | 11 (4 - 23) | 766 (98.8%) | 38 (27 - 61) | 202 (95.3%) | 10 (4 - 21) | 452 (96.6%) |
| Post-discharge Follow-up (days) | 856 (78 - 2002) | 534 (88.7%) | - | n.a. | 681 (11 - 1646) | 197 (92.9%) | - | n.a. |

| **Table S3** - Extracorporeal membrane oxygenation (ECMO) indication stratified by age and ECMO implantation timing. | | | | | | | | |
| --- | --- | --- | --- | --- | --- | --- | --- | --- |
|  | **Age Groups** | | | | | | | |
|  | **< 70 years (n=1376)** | | | | **≥ 70 years (n=680)** | | | |
|  | **ECMO implantation timing** | | | | **ECMO implantation timing** | | | |
|  | **Intra-operative (n=842)** | | **Post-operative (n=478)** | | **Intra-operative (n=407)** | | **Post-operative (n=258)** | |
| ECMO indication |  |  |  |  |  |  |  |  |
| Failure to wean | 503 | (59.7) | 0 | (0) | 260 | (63.9) | 0 | (0) |
| Acute pulmonary embolism | 0 | (0) | 2 | (0.4) | 1 | (0.2) | 0 | (0) |
| Arrhythmia | 10 | (1.2) | 23 | (4.8) | 3 | (0.7) | 7 | (2.7) |
| Cardiac arrest | 28 | (3.3) | 87 | (18.2) | 21 | (5.2) | 34 | (13.2) |
| Cardiogenic shock | 107 | (12.7) | 206 | (43.1) | 58 | (14.3) | 135 | (52.3) |
| Pulmonary hemorrhage | 4 | (0.5) | 1 | (0.2) | 3 | (0.7) | 1 | (0.4) |
| Right ventricular failure | 91 | (10.8) | 79 | (16.5) | 32 | (7.9) | 38 | (14.7) |
| Respiratory failure | 15 | (1.8) | 30 | (6.3) | 8 | (2.0) | 19 | (7.4) |
| Biventricular failure | 68 | (8.1) | 39 | (8.1) | 21 | (5.2) | 21 | (8.1) |
| Other | 16 | (1.9) | 11 | (2.3) | 0 | (0) | 3 | (1.2) |
| Post-operative implantation time (days) | n.a. | | 1 | (1-3) | n.a. | | 1 | (1-3) |
| Data are reported as n (% as valid percentage excluding missing values), or median (1^st^ and 3^rd^ quartile). | | | | | | | | |

***Sensitivity analysis after excluding patients operated between 2000 and 2010***

**Table S4-** Demographics and pre-operative characteristics of patients supported by post-cardiotomy ECMO after 2010.

| **Variable** | **Age group** | | **p value** |  |
| --- | --- | --- | --- | --- |
|  | **< 70 years**  **(n=1058)** | **≥ 70 years (n=547)** |  |  |
| Age (years) | 59 (51-65) | 74 (72-77.58) | <0.001 |  |
| Sex |  |  |  |  |
| Female | 389 (36.8) | 237 (43.3) |  |  |
| Male | 668 (36.2) | 310 (56.7) |  |  |
| Body mass index (kg/m2) | 26.32 (23.4-29.9) | 26 (23.8-30) | 0.47 |  |
| Hypertension | 603 (59.5) | 432 (82.1) | <0.001 |  |
| Dialysis | 102 (9.8) | 45 (8.5) | 0.385 |  |
| Myocardial infarction (last 30 days) | 100 (9.9) | 74 (14.1) | 0.013 |  |
| Smoking | 274 (29.1) | 94 (19.8) | <0.001 |  |
| Atrial fibrillation | 235 (22.2) | 183 (33.5) | <0.001 |  |
| Previous stroke | 137 (12.9) | 79 (14.4) | 0.406 |  |
| Diabetes mellitus | 236 (22.3) | 165 (30.2) | 0.001 |  |
| Chronic obstructive pulmonary disease | **88 (8.6)** | **57 (10.9)** | **0.143** |  |
| Peripheral artery disease | 127 (12) | 94 (17.2) | 0.004 |  |
| Pulmonary hypertension (>50 mmHg) | 182 (17.3) | 136 (25) | <0.001 |  |
| Previous cardiac surgery | 297 (28.1) | 114 (20.8) | 0.002 |  |
| Left ventricular ejection fraction (%) | 45 (29-60) | 50 (35-60) | <0.001 |  |
| Pre-operative creatinine (umol/L) | 99 (78-135) | 105.6 (83.1-141.4) | 0.39 |  |
| New York Heart Association class |  | | 0.065 |  |
| Class I | 88 (8.9) | 33 (6.2) |  |  |
| Class II | 219 (22.1) | 115 (21.5) |  |  |
| Class III | 368 (37.2) | 231 (43.2) |  |  |
| Class IV | 314 (31.7) | 156 (29.2) |  |  |
| Pre-operative condition |  | | |  |
| Cardiogenic shock | 228 (21.8) | 120 (22.3) | 0.817 |  |
| Intubation | 134 (12.7) | 50 (9.1) | 0.035 |  |
| Urgent surgery | **240 (22.9%)** | **106 (19.7%)** | **0.144** |  |
| Emergency surgery | 263 (25.2) | 138 (25.7) | 0.834 |  |
| Cardiac arrest | 76 (7.3) | 47 (8.7) | 0.318 |  |
| Vasopressors | 179 (17) | 64 (11.8) | 0.006 |  |
| Right ventricular failure | 90 (9.4) | 49 (9.4) | 0.992 |  |
| Preoperative diagnosis |  | | |  |
| Coronary artery disease | 453 (24.8) | 327 (59.8) | <0.001 |  |
| Aortic vessel disease | 209 (19.8) | 78 (14.3) | 0.006 |  |
| Aortic valve disease | 320 (30.2) | 223 (40.8) | <0.001 |  |
| Mitral valve disease | 328 (31) | 228 (41.7) | <0.001 |  |
| Tricuspid valve disease | 159 (15) | 118 (21.6) | 0.001 |  |
| Post-AMI ventricular septal rupture | 26 (2.5) | 18 (3.3) | 0.333 |  |
| Free wall/Papillary muscle rupture | 21 (2) | 6 (1.1) | 0.190 |  |
| Active endocarditis | **95 (9)** | **35 (6.4)** | **0.072** |  |
| Data are reported as n (% as valid percentage excluding missing values), or median (1^st^ and 3^rd^ quartile). P values by chi-square (for categorical data) or Student t-test (for parametric continuous data) and Mann–Whitney U test (for nonparametric continuous data) indicate statistically significant differences. Text in bold indicates differences compared with the overall population analysis. COPD, chronic obstructive pulmonary disease; AMI, acute myocardial infarction. | | | |  |
|  |  |  |  |  |
|  |  |  |  |  |

**Table S5 -** Procedural and extracorporeal membrane oxygenation (ECMO) characteristics of patients supported by post-cardiotomy ECMO after 2010.

| **Variable** | **Age group** | | **p value** |
| --- | --- | --- | --- |
|  | **< 70 years**  **(n=1058)** | **≥ 70 years**  **(n=547)** |  |
| Weight of surgery: |  |  | <0.001 |
| Unknown | 9 (0.9) | 0 (-) |  |
| Isolated CABG | 179 (16.9) | 109 (19.9) |  |
| Isolated non-CABG | 638 (60.3) | 268 (49) |  |
| 2 procedures | 45 (4.3) | 51 (9.3) |  |
| 3 or more procedures | 187 (17.7) | 119 (21.8) |  |
| CABG | 418 (39.5) | 299 (54.7) | <0.001 |
| Aortic valve surgery | 340 (32.1) | 224 (41) | <0.001 |
| Mitral valve surgery | 307 (29) | 207 (37.9) | <0.001 |
| Tricuspid valve surgery | 129 (12.2) | 100 (18.3) | 0.001 |
| Aortic surgery | 238 (22.5) | 87 (15.9) | 0.002 |
| Aortic surgery type |  |  |  |
| Aortic root  Ascending aorta and root  Ascending aorta  Ascending aorta and arch  Aortic arch and descending aorta | 36 (15.3)  67 (28.5)  54 (23)  64 (27.2)  14 (6) | 15 (17.4)  25 (29.1)  26 (30.2)  16 (18.6)  4 (4.7) |  |
| LVAD | **17 (1.6)** | **3 (0.5)** | **0.070** |
| Heart transplantation | 138 (13) | 2 (0.4) | <0.001 |
| Crossclamp time (min) | 104 (66-155) | 99 (68-142) | 0.39 |
| CPB time (min) | 211 (146.5-300) | 188 (130-267) | 0.001 |
| IABP implantation during hospital admission: |  |  | 0.116 |
| Pre-operative | 90 (31) | 34 (23.8) |  |
| Intra-operative | 200 (69) | 109 (76.2) |  |
| Left ventricular unloading | 283 (32.1) | 123 (27.9) | 0.12 |
| ECMO indication |  |  | 0.025 |
| Failure to wean | 363 (35.4) | 202 (37.5) |  |
| Acute pulmonary embolism | 2 (0.2) | 1 (0.2) |  |
| Arrhythmia | 26 (2.5) | 8 (1.5) |  |
| Cardiac arrest | 94 (9.2) | 42 (7.8) |  |
| Cardiogenic shock | 257 (25) | 168 (31.2) |  |
| Pulmonary hemorrhage | 5 (0.5) | 2 (0.4) |  |
| Right ventricular failure | 140 (13.6) | 59 (10.9) |  |
| Respiratory failure | 29 (2.8) | 20 (3.7) |  |
| Biventricular failrue | 87 (8.5) | 34 (6.3) |  |
| Other | 23 (2.2) | 3 (0.6) |  |
| ECMO implantation timing |  |  | 0.326 |
| Intra-operative | 661 (62.5) | 328 (60) |  |
| Post-operative | 397 (37.5) | 219 (40.0) |  |
| Cannulation approach |  |  | <0.001 |
| Unknown | 9 (0.9) | 24 (4.4) |  |
| Only central cannulation | 167 (15.8) | 78 (14.3) |  |
| Only peripheral cannulation | 508 (48) | 228 (41.7) |  |
| Mixed/switch cannulation | 374 (35.5) | 217 (39.7) |  |
| Open chest | 310 (42.6) | 176 (43.9) | 0.671 |
| ECMO duration (days) | 5 (2.6-8.9) | 4.9 (2.4-7.4) | 0.06 |
| Data are reported as n (% as valid percentage excluding missing values), or median (1^st^ and 3^rd^ quartile). P values by chi-square (for categorical data) or Student t-test (for parametric continuous data) and Mann–Whitney U test (for nonparametric continuous data) indicate statistically significant differences. Text in bold indicates differences compared with the overall population analysis. CABG. coronary artery bypass surgery. LVAD. left ventricle assist device. CPB. cardiopulmonary bypass. IABP. intra aortic balloon pump. ECMO. extracorporeal membrane oxygenation. | | | |

**Table S6-** Postoperative complications and mortality of patients supported by post-cardiotomy ECMO after 2010.

| **Variable** | **Age group** | | **p value** |
| --- | --- | --- | --- |
|  | **< 70 years (n=1058)** | **≥70 years**  **(n=547)** |  |
| Post-operative bleeding | 563 (54.6) | 313 (58.1) | 0.190 |
| Requiring re-thoracotomy | 369 (37.3) | 205 (40.2) | 0.276 |
| Cannulation site bleeding | 122 (11.8) | 68 (12.7) | 0.604 |
| Cerebral hemorrhage | 19 (1.9) | 13 (2.5) | 0.436 |
| Ischemic stroke | 124 (11.8) | 50 (9.2) | 0.112 |
| Leg ischemia | **100 (10)** | **38 (7.4)** | **0.095** |
| Bowel ischemia | 67 (6.9) | 19 (3.8) | 0.016 |
| Right ventricular failure | **174 (18.2)** | **110 (22.7)** | **0.040** |
| Acute kidney injury | 510 (52.3) | 275 (55.3) | 0.271 |
| Septic shock | 167 (17.5) | 76 (15.7) | 0.402 |
| ARDS | 45 (4.6) | 22 (4.4) | 0.848 |
| Multi-organ failure | 329 (31.4) | 207 (37.9) | 0.009 |
| In-hospital mortality | 579 (54.7) | 377 (68.9) | <0.001 |
| In-hospital mortality cause |  |  |  |
| Multi-organ failure | 207 (38.4) | 137 (39.7) |  |
| Sepsis | 39 (7.2) | 24 (7) |  |
| Persistent heart failure | 189 (35.1) | 123 (35.7) |  |
| Destributive shock syndrome | 11 (2) | 8 (2.3) |  |
| Bleeding | 32 (5.9) | 17 (4.9) |  |
| Neurological | 27 (5) | 16 (4.6) |  |
| Bowel ischemia | 14 (2.6) | 5 (1.4) |  |
| Other | 20 (3.7) | 15 (4.3) |  |
| Data are reported as n (% as valid percentage excluding missing values), or median (1^st^ and 3^rd^ quartile). P values by chi-square (for categorical data) or Student t-test (for parametric continuous data) and Mann–Whitney U test (for nonparametric continuous data) indicate statistically significant differences. Text in bold indicates differences compared with the overall population analysis. ARDS. acute respiratory distress syndrome. | | | |

***Subgroup analysis comparing in-hospital survivors and in-hospital non-survivors in patients ≥ 70 years old***

**Table S7-** Demographics and preoperative characteristics in patients ≥ 70 years old.

| **Variable** | **In-hospital mortality** | | **p value** |
| --- | --- | --- | --- |
|  | **No**  **(n=212)** | **Yes**  **(n=468)** |  |
| Age | 74.21 (72-78) | 74.05 (72-77.8) | 0.650 |
| Sex |  |  | 0.822 |
| Female | 90 (42.5) | 203 (43.4) |  |
| Male | 122 (57.5) | 265 (56.6) |  |
| Body mass index (kg/m2) | 26.03 (23.7-29.1) | 26.6 (23.8-30.1) | 0.30 |
| Hypertension | 165 (80.5) | 366 (81) | 0.884 |
| Dialysis | 20 (9.8) | 28 (6.3) | 0.113 |
| Myocardial infarction (last 30 days) | 29 (14.1) | 66 (14.6) | 0.878 |
| Smoking | 36 (21.3) | 80 (20.1) | 0.746 |
| Atrial fibrillation | 72 (34) | 159 (34) | 0.983 |
| Previous stroke | 22 (10.4) | 78 (16.7) | 0.032 |
| Diabetes mellitus | 64 (30.2) | 146 (31.2) | 0.792 |
| COPD | 23 (11.7) | 59 (13.1) | 0.627 |
| Peripheral artery disease | 42 (19.8) | 87 (18.6) | 0.707 |
| Pulmonary hypertension (>50 mmHg) | 52 (24.9) | 118 (25.3) | 0.915 |
| Previous cardiac surgery | 45 (21.2) | 104 (22.2) | 0.771 |
| Left ventricular ejection fraction (%) | 46 (35-60) | 50 (35-60) | 0.446 |
| Pre-operative creatinine (umol/L) | 99 (79.5-125) | 107 (85-150) | 0.011 |
| New York heart association class |  |  | 0.770 |
| Class I | 15 (7.2) | 25 (5.5) |  |
| Class II | 45 (21.6) | 95 (20.9) |  |
| Class III | 93 (44.7) | 202 (44.4) |  |
| Class IV | 55 (26.4) | 133 (29.2) |  |
| Pre-operative condition |  |  |  |
| Cardiogenic shock | 31 (15) | 109 (23.6) | 0.011 |
| Intubation | 15 (7.1) | 44 (9.4) | 0.318 |
| Urgent surgery | 43 (20.8) | 88 (19) | 0.585 |
| Emergency surgery | 46 (22.2) | 131 (28.2) | 0.103 |
| Cardiac arrest | 22 (10.5) | 47 (10.2) | 0.912 |
| Vasopressors | 25 (12) | 57 (12.2) | 0.921 |
| Right ventricular failure | 9 (4.9) | 46 (11.3) | 0.013 |
| Pre-operative diagnosis |  |  |  |
| Coronary artery disease | 127 (59.9) | 281 (60) | 0.973 |
| Aortic vessel disease | 25 (11.8) | 66 (14.1) | 0.412 |
| Aortic valve disease | 83 (39.2) | 200 (42.7) | 0.380 |
| Mitral valve disease | 81 (38.2) | 190 (40.6) | 0.555 |
| Tricuspid valve disease | 45 (21.2) | 88 (18.8) | 0.461 |
| Post-AMI ventricular septal rupture | 6 (2.8) | 16 (3.4) | 0.688 |
| Free wall/Papillary muscle rupture | 1 (0.5) | 7 (1.5) | 0.251 |
| Active endocarditis | 13 (6.1) | 25 (5.3) | 0.678 |
| Data are reported as n (% as valid percentage excluding missing values), or median (1^st^ and 3^rd^ quartile). P values by chi-square (for categorical data) or Student t-test (for parametric continuous data) and Mann–Whitney U test (for nonparametric continuous data) indicate statistically significant differences. COPD, chronic obstructive pulmonary disease; AMI, acute myocardial infarction. | | | |

**Table S8-** Procedural and extracorporeal membrane oxygenation (ECMO) characteristics in patients ≥ 70 years old.

| **Variable** | **In-hospital mortality** | | **p value** |
| --- | --- | --- | --- |
|  | **No**  **(n=212)** | **Yes**  **(n=468)** |  |
| Weight of surgery: |  |  | 0.084 |
| Unknown | 0 (-) | 0 (-) |  |
| Isolated CABG | 57 (26.9) | 87 (18.6) |  |
| Isolated non-CABG | 90 (42.5) | 235 (50.2) |  |
| 2 procedures | 22 (10.4) | 47 (10) |  |
| 3 or more procedures | 43 (20.3) | 99 (21.2) |  |
| CABG | 120 (56.6) | 253 (45.1) | 0.537 |
| Aortic valve surgery | 84 (39.6) | 196 (41.9) | 0.579 |
| Mitral valve surgery | 72 (34.1) | 174 (37.2) | 0.443 |
| Tricuspid valve surgery | 35 (16.5) | 76 (16.2) | 0.580 |
| Aortic surgery | 24 (11.3) | 77 (16.5) | 0.081 |
| Aortic surgery type |  |  |  |
| Aortic root  Ascending aorta and root  Ascending aorta  Ascending aorta and arch  Aortic arch and descending aorta | 5 (20.8)  8 (33.3)  6 (25.0)  4 (16.7)  1 (4.2) | 11 (14.5)  20 (26.3)  25 (32.9)  15 (19.7)  5 (6.6) |  |
| LVAD | 1 (0.5) | 2 (0.4) | 1.000 |
| Heart transplantation | 1 (0.5) | 3 (0.6) | 1.000 |
| Cross-clamp time (min) | 92 (61-131) | 100 (64-147) | 0.140 |
| CPB time (min) | 178 (120-243) | 199 (135-285) | 0.010 |
| IABP implantation during hospital admission | 54 (25.5) | 146 (31.6) | 0.106 |
| Pre-operative | 15 (27.8) | 38 (26) |  |
| Intra-operative | 39 (72.2) | 108 (74) |  |
| Left ventricular unloading | 41 (23.6) | 114 (30.6) | 0.087 |
| ECMO indication |  |  | 0.262 |
| Failure to wean | 76 (36.5) | 189 (40.9) |  |
| Acute pulmonary embolism | 0.00 | 1 (0.2) |  |
| Arrhythmia | 5 (2.4) | 5 (1.1) |  |
| Cardiac arrest | 19 (9.1) | 36 (7.8) |  |
| Cardiogenic shock | 59 (28.4) | 134 (29) |  |
| Pulmonary hemorrhage | 3 (1.4) | 1 (0.2) |  |
| Right ventricular failure | 24 (11.5) | 46 (10) |  |
| Respiratory failure | 12 (5.8) | 15 (3.2) |  |
| Biventricular failure | 9 (4.3) | 33 (7.1) |  |
| Other | 1 (0.5) | 2 (0.4) |  |
| Cannulation approach |  |  | <0.039 |
| Unknown | 13 (6.1) | 17 (3.6) |  |
| Only central cannulation | 23 (10.8) | 84 (17.9) |  |
| Only peripheral cannulation | 100 (47.2) | 190 (40.6) |  |
| Mixed/switch cannulation | 76 (35.8) | 177 (37.8) |  |
| ECMO implant timing |  |  | 0.766 |
| Intra-operative | 127 (59.9) | 286 (61.1) |  |
| Post-operative | 85 (40.1) | 182 (38.9) |  |
| Open chest | 63 (41.7) | 168 (47.2) | 0.258 |
| ECMO duration (days) | 4.73 (3-6.61) | 4.9 (2-8) | 0.551 |
| Data are reported as n (% as valid percentage excluding missing values), or median (1^st^ and 3^rd^ quartile). P values by chi-square (for categorical data) or Student t-test (for parametric continuous data) and Mann–Whitney U test (for nonparametric continuous data) indicate statistically significant differences. CABG. coronary artery bypass surgery. LVAD. left ventricle assist device. CPB. cardiopulmonary bypass. IABP. intra aortic balloon pump. ECMO. extracorporeal membrane oxygenation. | | | |

**Table S9-** Postoperative complications in patients ≥ 70 years old.

| **Variable** | **In-hospital mortality** | | | **Variable** |
| --- | --- | --- | --- | --- |
|  | **No**  **(n=212)** | | **Yes**  **(n=468)** |  |
| Post-operative bleeding | 102 (48.8) | 293 (63.3) | | <0.001 |
| Requiring re-thoracotomy | 70 (36.6) | 195 (44.1) | | 0.080 |
| Cannulation site bleeding | 24 (11.5) | 62 (13.4) | | 0.494 |
| Cerebral hemorrhage | 4 (2.1) | 13 (2.9) | | 0.529 |
| Ischemic stroke | 30 (14.2) | 34 (7.3) | | 0.005 |
| Leg ischemia | 15 (7.5) | 33 (7.6) | | 0.968 |
| Bowel ischemia | 2 (1) | 22 (5.1) | | 0.015 |
| Right ventricular failure | 30 (16.4) | 110 (26.4) | | 0.008 |
| Acute kidney injury | 106 (56.4) | 255 (59.6) | | 0.458 |
| Septic shock | 22 (12) | 71 (17.1) | | 0.116 |
| ARDS | 8 (4.2) | 25 (5.9) | | 0.395 |
| Multi-organ failure | 11 (5.2) | 243 (52.5) | | <0.001 |
| In-hospital mortality cause |  |  | |  |
| Multi-organ failure |  | 160 (37) | |  |
| Sepsis |  | 29 (6.7) | |  |
| Persistent heart failure |  | 160 (37) | |  |
| Destributive shock syndrome |  | 8 (1.9) | |  |
| Bleeding |  | 21 (4.9) | |  |
| Neurological |  | 25 (5.8) | |  |
| Bowel ischemia |  | 7 (1.6) | |  |
| Other |  | 22 (5.1) | |  |
| Data are reported as n (% as valid percentage excluding missing values), or median (1^st^ and 3^rd^ quartile). P values by chi-square (for categorical data) or Student t-test (for parametric continuous data) and Mann–Whitney U test (for nonparametric continuous data) indicate statistically significant differences. ARDS. acute respiratory distress syndrome. | | | | |

**Table S10 -** Mixed Cox proportional hazards for in-hospital mortality.

|  | **Hazard Ratio** | **95% CI** | | **P value** |  |
| --- | --- | --- | --- | --- | --- |
|  |  | **Lower limit** | **Upper limit** |  |  |
| **Model 1: crude model** | | | | |  |
| Age | 1.02 | 0.99 | 1.04 | 0.089 |  |
| Sex-female | 0.99 | 0.82 | 1.20 | 0.94 |  |
| **Model 2: demographic data and preoperative variables** | | | | |  |
| **Age** | **1.03** | **1.00** | **1.06** | **0.02** |  |
| Dialysis | 0.77 | 0.51 | 1.15 | 0.20 |  |
| Myocardial infarction | 0.86 | 0.68 | 1.08 | 0.20 |  |
| **Stroke** | **1.41** | **1.08** | **1.85** | **0.01** |  |
| Atrial fibrillation | 1.01 | 0.83 | 1.24 | 0.91 |  |
| Diabetes mellitus | 0.91 | 0.73 | 1.11 | 0.35 |  |
| COPD | 1.14 | 0.86 | 1.51 | 0.36 |  |
| Peripheral artery disease | 1.08 | 0.84 | 1.38 | 0.54 |  |
| Pulmonary hypertension | 1.00 | 0.79 | 1.27 | 0.98 |  |
| Previous cardiac surgery | 1.02 | 0.81 | 1.28 | 0.88 |  |
| Preoperative left ventricular ejection fraction | 1.00 | 1.00 | 1.01 | 0.59 |  |
| Preoperative cardiogenic shock | 1.17 | 0.89 | 1.52 | 0.26 |  |
| Emergency surgery | 1.21 | 0.95 | 1.55 | 0.13 |  |
| Preoperative cardiac arrest | 1.02 | 0.73 | 1.41 | 0.93 |  |
| **Preoperative right ventricular failure** | **1.51** | **1.08** | **2.10** | **0.02** |  |
| **Model 3: demographic data and preoperative and intraoperative variables** | | | | |  |
| Sex-female | 1.01 | 0.83 | 1.23 | 0.89 |  |
| **Age** | **1.03** | **1.00** | **1.06** | **0.02** |  |
| Dialysis | 0.78 | 0.52 | 1.18 | 0.24 |  |
| Myocardial infarction | 0.90 | 0.71 | 1.14 | 0.39 |  |
| **Stroke** | **1.42** | **1.09** | **1.87** | **0.01** |  |
| Atrial fibrillation | 1.02 | 0.83 | 1.26 | 0.85 |  |
| Diabetes mellitus | 0.94 | 0.76 | 1.16 | 0.56 |  |
| COPD | 1.12 | 0.84 | 1.48 | 0.45 |  |
| Peripheral artery disease | 1.07 | 0.83 | 1.37 | 0.61 |  |
| Pulmonary hypertension | 1.03 | 0.81 | 1.31 | 0.78 |  |
| Previous cardiac surgery | 0.94 | 0.73 | 1.20 | 0.60 |  |
| Preoperative left ventricular ejection fraction | 1.00 | 0.99 | 1.01 | 0.74 |  |
| Preoperative cardiogenic shock | 1.16 | 0.89 | 1.52 | 0.27 |  |
| Emergency surgery | 1.17 | 0.91 | 1.51 | 0.22 |  |
| Preoperative cardiac arrest | 1.08 | 0.77 | 1.50 | 0.67 |  |
| **Preoperative right ventricular failure** | **1.56** | **1.11** | **2.19** | **0.01** |  |
| CPB time | 1.00 | 1.00 | 1.00 | 0.23 |  |
| CABG | 1.03 | 0.83 | 1.28 | 0.77 |  |
| Aortic valve surgery | 1.03 | 0.83 | 1.27 | 0.81 |  |
| Mitral valve surgery | 1.03 | 0.81 | 1.30 | 0.80 |  |
| Tricuspid valve surgery | 0.96 | 0.72 | 1.27 | 0.76 |  |
| **Aortic surgery** | **1.55** | **1.17** | **2.06** | **<0.001** |  |
| **Model 4: demographic data and preoperative, intraoperative, and ECMO variables** | | | | |  |
| Sex-female | 1.00 | 0.83 | 1.22 | 0.95 |  |
| **Age** | **1.03** | **1.00** | **1.06** | **0.03** |  |
| Dialysis | 0.78 | 0.52 | 1.19 | 0.25 |  |
| Myocardial infarction | 0.90 | 0.71 | 1.15 | 0.41 |  |
| **Stroke** | **1.41** | **1.07** | **1.84** | **0.01** |  |
| Atrial fibrillation | 1.04 | 0.84 | 1.29 | 0.74 |  |
| Diabetes mellitus | 0.94 | 0.76 | 1.16 | 0.54 |  |
| COPD | 1.10 | 0.82 | 1.46 | 0.53 |  |
| Peripheral artery disease | 1.06 | 0.82 | 1.37 | 0.65 |  |
| Pulmonary hypertension | 1.03 | 0.81 | 1.32 | 0.79 |  |
| Previous cardiac surgery | 0.94 | 0.73 | 1.20 | 0.60 |  |
| Preoperative left ventricular ejection fraction | 1.00 | 0.99 | 1.01 | 0.65 |  |
| Preoperative cardiogenic shock | 1.17 | 0.89 | 1.53 | 0.26 |  |
| Emergency surgery | 1.13 | 0.87 | 1.47 | 0.37 |  |
| Preoperative cardiac arrest | 1.04 | 0.73 | 1.49 | 0.82 |  |
| **Preoperative right ventricular failure** | **1.57** | **1.12** | **2.22** | **0.01** |  |
| CPB time | 1.00 | 1.00 | 1.00 | 0.14 |  |
| CABG | 1.02 | 0.82 | 1.28 | 0.83 |  |
| Aortic valve surgery | 1.02 | 0.82 | 1.27 | 0.88 |  |
| Mitral valve surgery | 0.99 | 0.78 | 1.25 | 0.93 |  |
| Tricuspid valve surgery | 0.96 | 0.72 | 1.28 | 0.76 |  |
| **Aortic surgery** | **1.55** | **1.16** | **2.06** | **<0.001** |  |
| ECMO Indication: |  |  |  |  |  |
| Acute pulmonary embolism | 2.09 | 0.01 | 518.99 | 0.76 |  |
| Arrhythmia | 0.71 | 0.27 | 1.85 | 0.48 |  |
| Cardiac arrest | 0.98 | 0.63 | 1.51 | 0.91 |  |
| Cardiogenic shock | 0.91 | 0.67 | 1.24 | 0.56 |  |
| Pulmonary hemorrhage | 0.19 | 0.03 | 1.40 | 0.10 |  |
| Right ventricular failure | 0.78 | 0.54 | 1.13 | 0.18 |  |
| Respiratory failure | 0.67 | 0.38 | 1.19 | 0.17 |  |
| Biventricular failure | 1.11 | 0.71 | 1.74 | 0.65 |  |
| Other | 0.68 | 0.15 | 3.08 | 0.61 |  |
| ECMO implant timing: |  |  |  |  |  |
| Intensive care unit | 1.09 | 0.83 | 1.43 | 0.53 |  |
| Ward | 1.12 | 0.50 | 2.51 | 0.77 |  |
| Cath lab | 1.74 | 0.58 | 5.21 | 0.32 |  |
| **Model 5: demographic data, preoperative, intraoperative, and postoperative complications** | | | | |  |
| Sex-female | 1.03 | 0.85 | 1.25 | 0.75 |  |
| **Age** | **1.03** | **1.00** | **1.06** | **0.04** |  |
| Dialysis | 0.85 | 0.56 | 1.28 | 0.44 |  |
| Myocardial infarction | 0.91 | 0.71 | 1.16 | 0.44 |  |
| **Stroke** | **1.40** | **1.06** | **1.84** | **0.02** |  |
| Atrial fibrillation | 1.01 | 0.82 | 1.25 | 0.92 |  |
| Diabetes mellitus | 0.91 | 0.74 | 1.13 | 0.41 |  |
| COPD | 1.09 | 0.82 | 1.46 | 0.55 |  |
| Peripheral artery disease | 1.04 | 0.80 | 1.34 | 0.78 |  |
| Pulmonary hypertension | 1.03 | 0.81 | 1.31 | 0.82 |  |
| Previous cardiac surgery | 1.01 | 0.78 | 1.29 | 0.96 |  |
| Preoperative left ventricular ejection fraction | 1.00 | 0.99 | 1.01 | 0.74 |  |
| Preoperative cardiogenic shock | 1.26 | 0.95 | 1.68 | 0.10 |  |
| Emergency surgery | 1.14 | 0.88 | 1.47 | 0.33 |  |
| Preoperative cardiac arrest | 1.12 | 0.80 | 1.56 | 0.52 |  |
| **Preoperative right ventricular failure** | **1.45** | **1.02** | **2.07** | **0.04** |  |
| CPB time | 1.00 | 1.00 | 1.00 | 0.10 |  |
| CABG | 1.05 | 0.84 | 1.30 | 0.69 |  |
| Aortic valve surgery | 0.95 | 0.76 | 1.19 | 0.66 |  |
| Mitral valve surgery | 1.07 | 0.84 | 1.37 | 0.60 |  |
| Tricuspid valve surgery | 0.86 | 0.64 | 1.14 | 0.29 |  |
| **Aortic surgery** | **1.65** | **1.24** | **2.20** | **<0.001** |  |
| **Postoperative bleeding** | **1.24** | **1.01** | **1.54** | **0.04** |  |
| **Postoperative stroke** | **0.48** | **0.33** | **0.70** | **<0.001** |  |
| **Postoperative cardiac arrest** | **1.65** | **1.29** | **2.10** | **<0.001** |  |
| Postoperative bowel ischemia | 1.11 | 0.69 | 1.79 | 0.66 |  |
| Postoperative acute kidney injury | 0.94 | 0.76 | 1.16 | 0.55 |  |
| **Postoperative septic shock** | **0.66** | **0.50** | **0.86** | **<0.001** |  |
| **Postoperative right ventricular failure** | **1.29** | **1.01** | **1.64** | **0.04** |  |
| COPD. chronic obstructive pulmonary disease. CPB. cardiopulmonary bypass. CABG. coronary artery bypass graft. ECMO. Extracorporeal membrane oxygenation. | | | | |  |
|  |  |  |  |  |  |

***Subgroup analysis of patients ≥ 80 years old***

**Table S11 -** Demographics and preoperative characteristics of the octogenarian population (≥ 80 years old).

| **Variable** | **n=91** |
| --- | --- |
| Age | 82 (80.4-83) |
| Sex |  |
| Female | 44 (48.3) |
| Male | 47 (51.6) |
| Body mass index (kg/m2) | 25.8 (23.4-29.3) |
| Hypertension | 74 (82.2) |
| Dialysis | 4 (4.7) |
| Myocardial infarction (last 30 days) | 14 (15.5) |
| Smoking | 8 (11.4) |
| Atrial fibrillation | 32 (35.1) |
| Previous stroke | 8 (8.7) |
| Diabetes mellitus | 18 (19.7) |
| COPD | 7 (8.3) |
| Peripheral artery disease | 15 (16.4) |
| Pulmonary hypertension (>50 mmHg) | 31 (34.4) |
| Previous cardiac surgery | 23 (25.2) |
| Left ventricular ejecion fraction (%) | 45 (35-60) |
| Pre-operative creatinine (umol/L) | 141.1 (1.11-539.4) |
| New York heart association class |  |
| Class I | 5 (5.6) |
| Class II | 21 (23.8) |
| Class III | 40 (45.4) |
| Class IV | 22 (25) |
| Pre-operative condition |  |
| Cardiogenic shock | 14 (15.7) |
| Intubation | 6 (6.5) |
| Urgent surgery | 18 (20.2) |
| Emergency surgery | 20 (22.4) |
| Cardiac arrest | 12 (13.3) |
| Vasopressors | 8 (8.8) |
| Right ventricular failure | 6 (8.4) |
| Pre-operative diagnosis |  |
| Coronary artery disease | 53 (58.2) |
| Aortic vessel disease | 16 (17.5) |
| Aortic valve disease | 51 (56) |
| Mitral valve disease | 31 (34) |
| Tricuspid valve disease | 20 (21.9) |
| Post-AMI ventricular septal rupture | 2 (2.1) |
| Free wall/Papillary mucle rupture | 0 (0) |
| Active endocarditis | 4 (4.3) |
| Data are reported as n (% as valid percentage, excluding missing values) or median (1^st^ and 3^rd^ quartile). COPD. chronic obstructive pulmonary disease. AMI. acute myocardial infarction. | |

**Table S12 -** Procedural and ECMO characteristics of the octogenarian population (≥ 80 years old).

| **Variable** | **n=91** |
| --- | --- |
| Weight of surgery: |  |
| Unknown | 0 (-) |
| Isolated CABG | 16 (17.5) |
| Isolated non-CABG | 32 (35.1) |
| 2 procedures | 20 (21.9) |
| 3 or more procedures | 23 (25.2) |
| CABG | 53 (58.2) |
| Aortic valve surgery | 47 (51.6) |
| Mitral valve surgery | 30 (32.9) |
| Tricuspid valve surgery | 19 (20.8) |
| Aortic surgery | 17 (18.6) |
| Aortic surgery type  Aortic root  Ascending aorta and root  Ascending aorta  Ascending aorta and arch  Aortic arch and descending aorta | 4 (23.5)  3 (17.6)  7 (41.2)  3 (17.6)  0 (0.0) |
| LVAD | 0 (0) |
| Heart transplantation | 0 (0) |
| Cross-clamp time (min) | 105 (73-144) |
| CPB time (min) | 192 (135-275) |
| IABP implantation during hospital admission |  |
| Pre-operative | 5 (22.7) |
| Intra-operative | 17 (77.2) |
| Left ventriculat unloading | 18 (23.3) |
| ECMO indication |  |
| Failure to wean | 43 (49.4) |
| Acute pulmonary embolism | 0 (0) |
| Arrhythmia | 0 (0) |
| Cardiac arrest | 8 (9.1) |
| Cardiogenic shock | 17 (19.5) |
| Pulmonary hemorrhage | 0 (0) |
| Right ventricular failure | 9 (10.3) |
| Respiratory failure | 4 (4.5) |
| Biventricular failrue | 6 (6.8) |
| Other | 0 (0) |
| ECMO implant timing |  |
| Intra-operative | 59 (64.8) |
| Post-operative | 32 (35.2) |
| Cannulation approach |  |
| Unknown | 2 (2.2) |
| Only central cannulation | 15 (16.5) |
| Only peripheral cannulation | 34 (37.4) |
| Mixed/switch cannulation | 40 (44) |
| Chest ststus: open | 35 (52.2) |
| ECMO duration (days) | 3.5 (1.5-6) |
| Data are reported as n (% as valid percentage, excluding missing values) or median (1^st^ and 3^rd^ quartile). CABG. coronary artery bypass surgery. LVAD. left ventricle assist device. CPB. cardiopulmonary bypass. IABP. intra aortic balloon pump. ECMO. extra-corporeal membrane oxygenation. | |

**Table S13 -** Mortality and postoperative complications in the octogenarian population (≥ 80 years old).

| **Variable** | **n=91** |
| --- | --- |
| Post-operative bleeding | 46 (52.3) |
| Requiring re-thoracotomy | 30 (38.5) |
| Cannulation site bleeding | 10 (11.5) |
| Cerebral hemorrhage | 1 (1.2) |
| Ischemic stroke | 6 (6.6) |
| Leg ischemia | 4 (4.7) |
| Bowel ischemia | 5 (6.3) |
| Right ventricular failure | 16 (21.9) |
| Acute kidney injury | 42 (53.8) |
| Septic shock | 8 (11) |
| ARDS | 1 (1.3) |
| Multi-organ failure | 33 (36.3) |
| In-hospital mortality | 64 (70.3) |
| In-hospital mortality cause |  |
| Multi-organ failure | 20 (35.1) |
| Sepsis | 4 (7) |
| Persistent heart failure | 21 (36.8) |
| Destributive shock syndrome | 2 (3.5) |
| Bleeding | 2 (3.5) |
| Neurological | 3 (5.3) |
| Bowel ischemia | 2 (3.5) |
| Other | 3 (5.3) |
| Data are reported as n (% as valid percentage, excluding missing values) or median (1^st^ and 3^rd^ quartile). ARDS. acute respiratory distress syndrome. | |

**Supplemental Figures**

**Figure S1-** Flow-chart describing patients included in the current study.


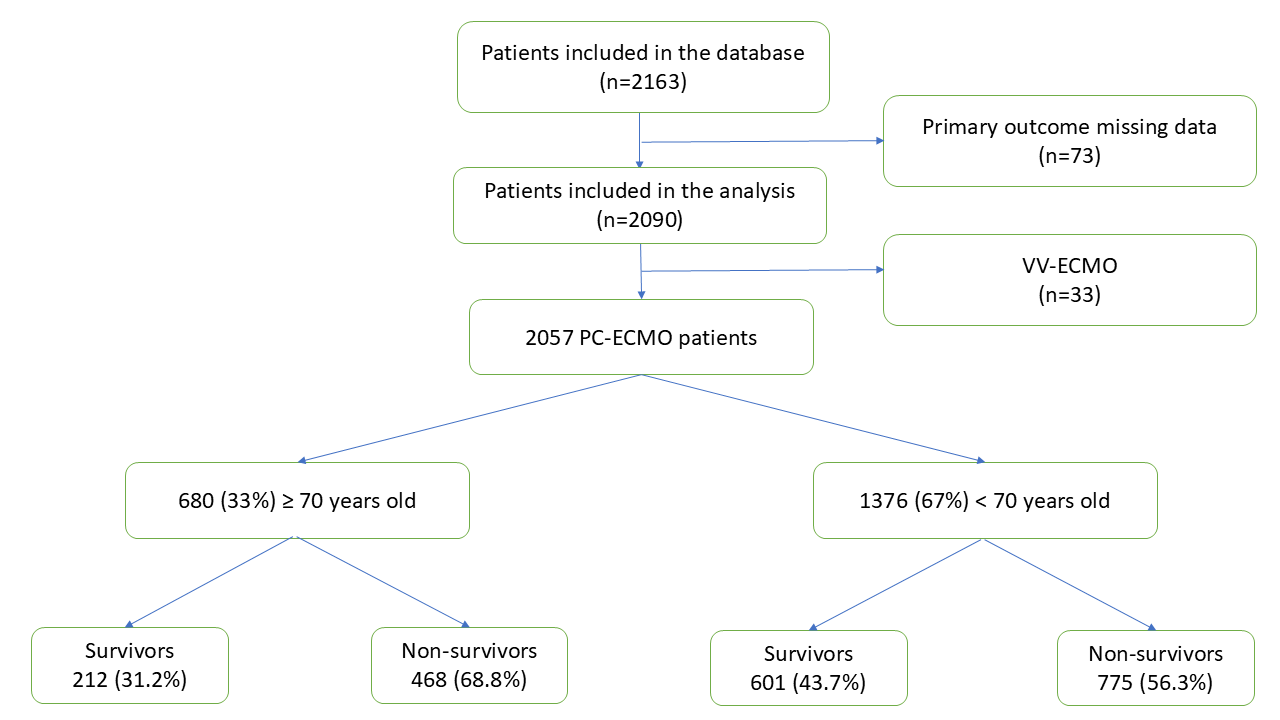


**Figure S2 -** Post-discharge survival in patients supported with post-cardiotomy ECMO after 2010 and discharged alive as represented by Kaplan Mayer curve with 95% confidence intervals.

**
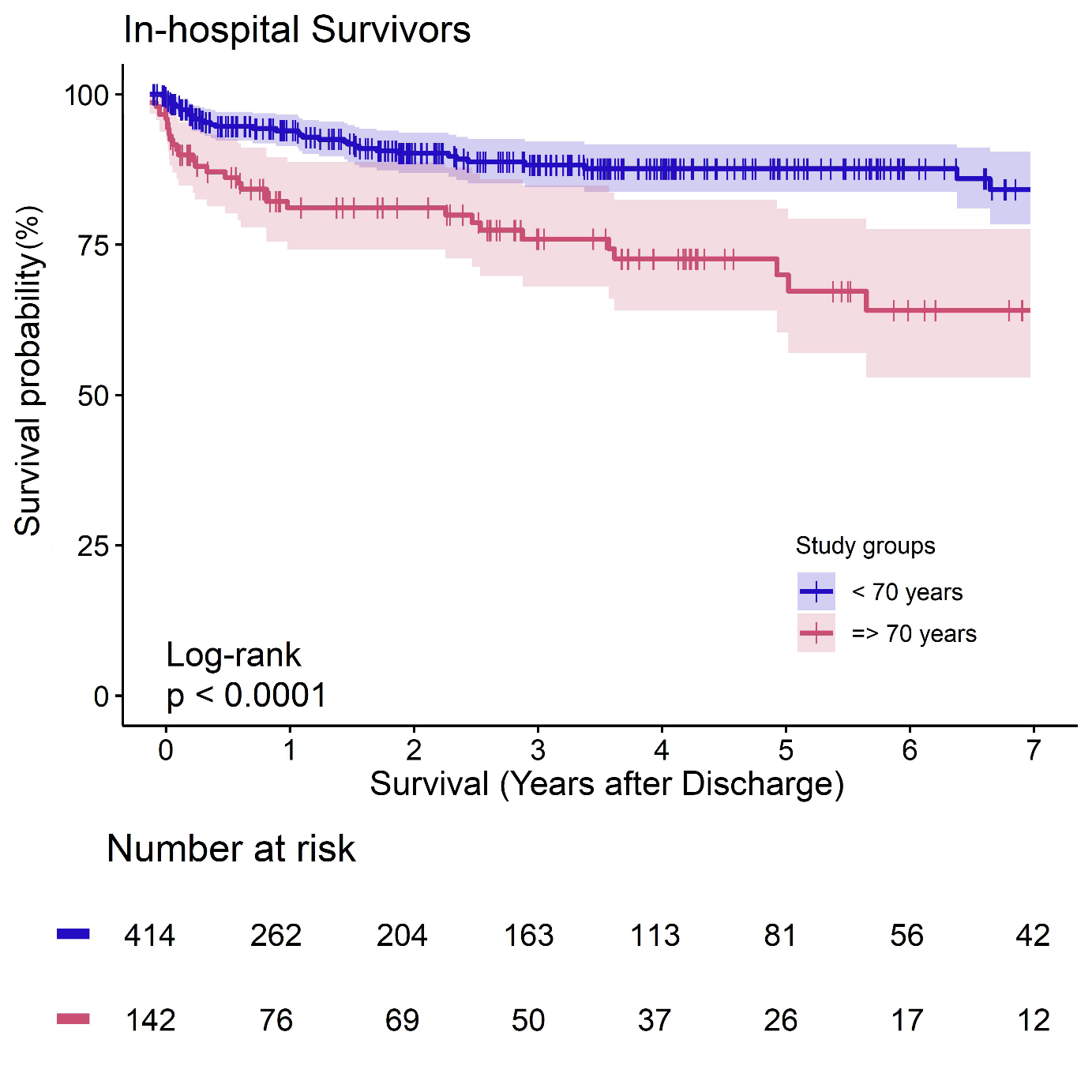
**

**Figure S3 –** Relationship between age and in-hospital mortality by restricted cubic spline plot. Knots were located at age values of 70, 73, 76 and 82 years.

**
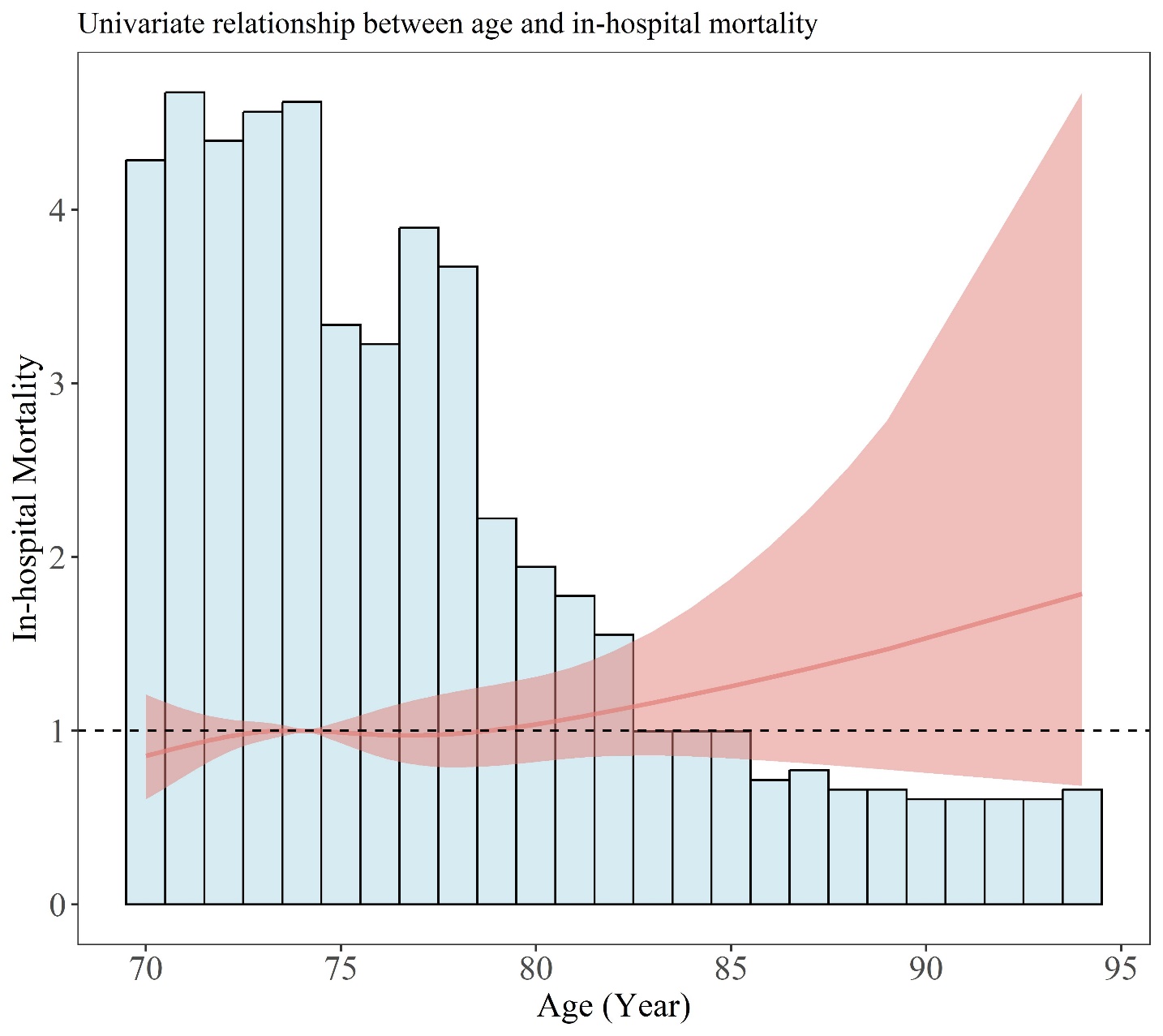
**

**Figure S4 -** Post-discharge survival in the octogenarian population (≥ 80 years old) as represented by Kaplan Mayer curve with 95% confidence intervals.


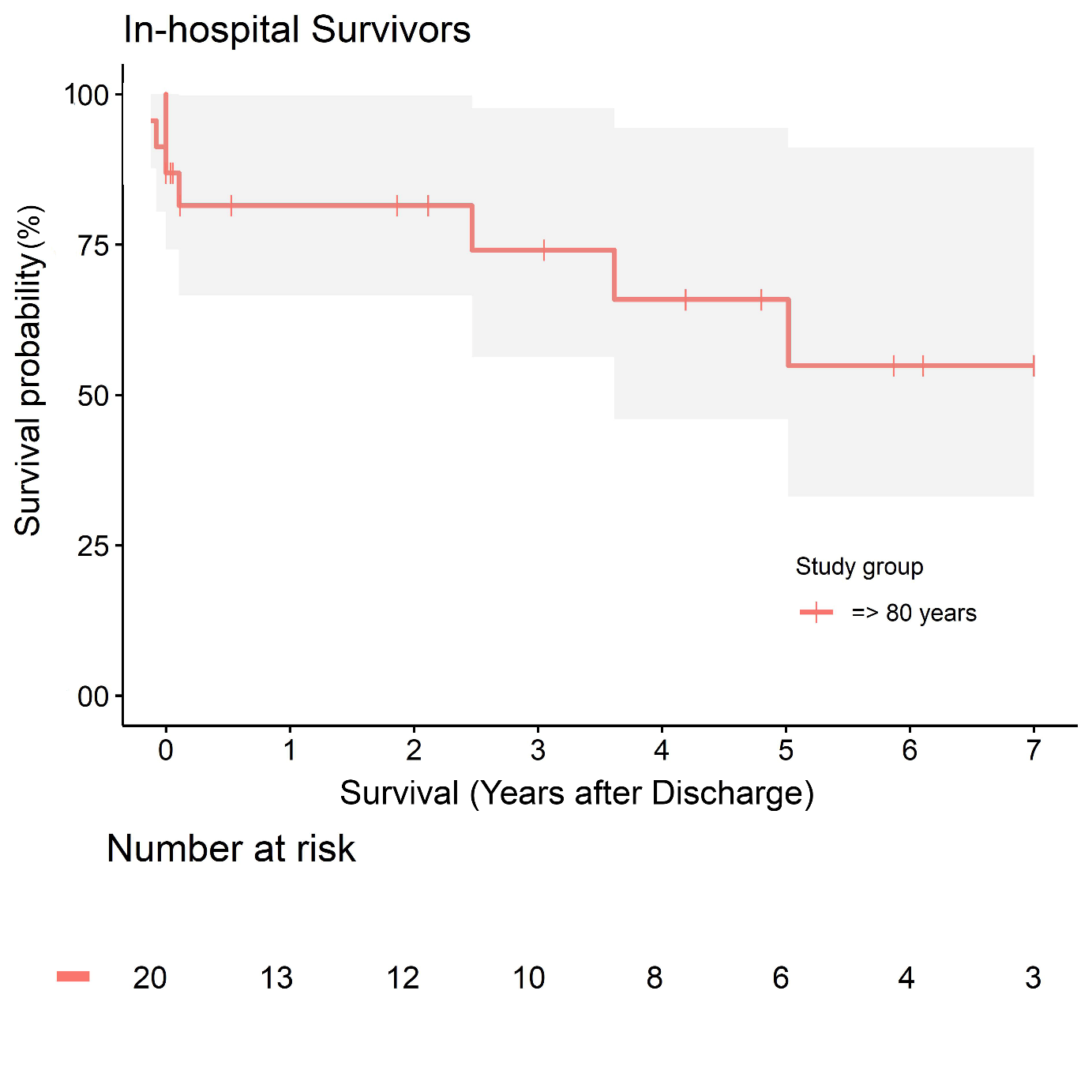


**Supplemental References**

1. Heidari S, Babor TF, De Castro P, Tort S, Curno M. Sex and Gender Equity in Research: rationale for the SAGER guidelines and recommended use. *Res Integr Peer Rev*. 2016;1:2. doi:10.1186/s41073-016-0007-6

2. Richie C. Sex, not gender. A plea for accuracy. *Exp Mol Med*. Nov 8 2019;51(11):1. doi:10.1038/s12276-019-0341-0

3. Clayton JA, Tannenbaum C. Reporting Sex, Gender, or Both in Clinical Research? *Jama*. Nov 8 2016;316(18):1863-1864. doi:10.1001/jama.2016.16405

4. Williams B, Mancia G, Spiering W, et al. 2018 ESC/ESH Guidelines for the management of arterial hypertension. *Eur Heart J*. Sep 1 2018;39(33):3021-3104. doi:10.1093/eurheartj/ehy339

5. Global Initiative for Chronic Obstructive Lung Disease (GOLD). Global strategy for the diagnosis, management, and prevention of chronic obstructive pulmonary disease: 2022 report

6. Nashef SA, Roques F, Sharples LD, et al. EuroSCORE II. *Eur J Cardiothorac Surg*. Apr 2012;41(4):734-44; discussion 744-5. doi:10.1093/ejcts/ezs043

7. McDonagh TA, Metra M, Adamo M, et al. 2021 ESC Guidelines for the diagnosis and treatment of acute and chronic heart failure. *Eur Heart J*. Sep 21 2021;42(36):3599-3726. doi:10.1093/eurheartj/ehab368

8. Singer M, Deutschman CS, Seymour CW, et al. The Third International Consensus Definitions for Sepsis and Septic Shock (Sepsis-3). *Jama*. Feb 23 2016;315(8):801-10. doi:10.1001/jama.2016.0287

9. Gorter TM, van Veldhuisen DJ, Bauersachs J, et al. Right heart dysfunction and failure in heart failure with preserved ejection fraction: mechanisms and management. Position statement on behalf of the Heart Failure Association of the European Society of Cardiology. *Eur J Heart Fail*. Jan 2018;20(1):16-37. doi:10.1002/ejhf.1029

10. Bozkurt B, Coats AJS, Tsutsui H, et al. Universal definition and classification of heart failure: a report of the Heart Failure Society of America, Heart Failure Association of the European Society of Cardiology, Japanese Heart Failure Society and Writing Committee of the Universal Definition of Heart Failure: Endorsed by the Canadian Heart Failure Society, Heart Failure Association of India, Cardiac Society of Australia and New Zealand, and Chinese Heart Failure Association. *Eur J Heart Fail*. Mar 2021;23(3):352-380. doi:10.1002/ejhf.2115

11. Easton JD, Saver JL, Albers GW, et al. Definition and evaluation of transient ischemic attack: a scientific statement for healthcare professionals from the American Heart Association/American Stroke Association Stroke Council; Council on Cardiovascular Surgery and Anesthesia; Council on Cardiovascular Radiology and Intervention; Council on Cardiovascular Nursing; and the Interdisciplinary Council on Peripheral Vascular Disease. The American Academy of Neurology affirms the value of this statement as an educational tool for neurologists. *Stroke*. Jun 2009;40(6):2276-93. doi:10.1161/STROKEAHA.108.192218

12. Shanmugam G. Vasoplegic syndrome--the role of methylene blue. *Eur J Cardiothorac Surg*. Nov 2005;28(5):705-10. doi:10.1016/j.ejcts.2005.07.011

13. Mariani S, Bari G, Ravaux JM, et al. Heterogeneity in Clinical Practices for Post-Cardiotomy Extracorporeal Life Support: a Pilot Survey from the PELS-1 Multicenter Study. *Artif Organs*. Jun 23 2023;doi:10.1111/aor.14601

14. Mariani S, Heuts S, van Bussel BCT, et al. Patient and Management Variables Associated With Survival After Postcardiotomy Extracorporeal Membrane Oxygenation in Adults: The PELS-1 Multicenter Cohort Study. *Journal of the American Heart Association*. Jul 18 2023;12(14):e029609. doi:10.1161/jaha.123.029609
